# Platelets Recognize Brain-Specific Glycolipid Structures, Respond to Neurovascular Damage and Promote Neuroinflammation

**DOI:** 10.1371/journal.pone.0058979

**Published:** 2013-03-26

**Authors:** Ilya Sotnikov, Tatyana Veremeyko, Sarah C. Starossom, Natalia Barteneva, Howard L. Weiner, Eugene D. Ponomarev

**Affiliations:** 1 Center for Neurologic Diseases, Brigham and Women’s Hospital, Harvard Medical School, Boston, Massachusetts, United States of America; 2 Division of Neonatal-Perinatal Medicine, Emory University School of Medicine, Atlanta, Georgia, United States of America; 3 The Immune Disease Institute, Harvard Medical School, Boston, Massachusetts, United States of America; 4 School for Biomedical Sciences, The Chinese University of Hong Kong, Shatin, NT, Hong Kong; Virginia Commonwealth University, United States of America

## Abstract

Platelets respond to vascular damage and contribute to inflammation, but their role in the neurodegenerative diseases is unknown. We found that the systemic administration of brain lipid rafts induced a massive platelet activation and degranulation resulting in a life-threatening anaphylactic-like response in mice. Platelets were engaged by the sialated glycosphingolipids (gangliosides) integrated in the rigid structures of astroglial and neuronal lipid rafts. The brain-abundant gangliosides GT1b and GQ1b were specifically recognized by the platelets and this recognition involved multiple receptors with P-selectin (CD62P) playing the central role. During the neuroinflammation, platelets accumulated in the central nervous system parenchyma, acquired an activated phenotype and secreted proinflammatory factors, thereby triggering immune response cascades. This study determines a new role of platelets which directly recognize a neuronal damage and communicate with the cells of the immune system in the pathogenesis of neurodegenerative diseases.

## Introduction

The inflammation in the central nervous system (CNS) is a complex and understudied process, underlying numerous nosologies with a high socioeconomic impact worldwide. With the current focus on the disease prevention it is of vital importance to dissect the initiation of a neuronal damage and define the specific triggers and first-line responders. Platelets, or thrombocytes, are small (2–3 µm in diameter) non-nucleated cells produced by megakaryocytes through a budding process which takes place primarily in the bone marrow [1], [2]. In the peripheral blood platelets outnumber leukocytes almost 100-fold and are known to play a pivotal role in thrombosis and hemostasis in response to a blood vessel injury. There is a growing body of knowledge demonstrating that platelets contribute to the inflammation in a number of pathologic processes including infection, atherosclerosis, and cancer metastasis [3]–[7]. The important role of platelets was reported in the pathogenesis of arthritis where they recognize the exposed collagen and produce microparticles upon activation [8]. The platelets have also been reported as the first line of defense against blood-born pathogens such as *Listeria monocytogenes* [9]. A number of molecular structures which are exposed to the blood stream after the disruption of the endothelial cell integrity are recognized by platelets. These structures include von Willebrand factor, collagen, laminin, vitronectin and fibrinogen. A number of specific receptors expressed by platelets including CD42, and integrins consisting of various α light chains and β1 and β3 heavy chains are operative in the recognition of these factors [10].

The role of platelets is well established in cardiovascular pathologies and cancer but it is poorly understood in neurologic diseases [11], [12]. The activation of platelets has been reported in Alzheimer’s disease (AD) and multiple sclerosis (MS) [13]–[17], but little is known about the details of such activation. The platelet activation during the neuroinflammation was associated with the interaction between platelets and damaged endothelial cells and leukocytes [18], [19]. The presence of specific structures in the CNS, which can be directly recognized by platelets, has not been reported.

In contrast to other tissues, the CNS is separated from the rest of the body by the blood brain barrier (BBB) made of the tight junctions of brain endothelial and astroglial cells [20]. Neuronal and astroglial cells are functionally integrated with vascular endothelial cells into so called “neurovascular units” [20]–[22], and disruption of the the BBB is a hallmark of many neurodegenerative and neuroinflammatory diseases, including MS [23]. In our work we focused on the roles of the neurovascular unit damage and mechanisms of the resultant platelet activation. Our analysis revealed that platelets recognize the sialated gangliosides in the lipid rafts on the surface of astroglial and neuronal cells, which was important for the development of the experimental autoimmune encephalomyelitis (EAE), an animal model of neuroinflammatory and neurodegenerative disease such as MS. This study presents a significant step forward in understanding the new types of the neuronal damage signals that involve platelets as effectors.

## Results

### The Brain Lipid Rafts Induce a Platelet-mediated Anaphylactic-like Reaction

**Figure 1:**
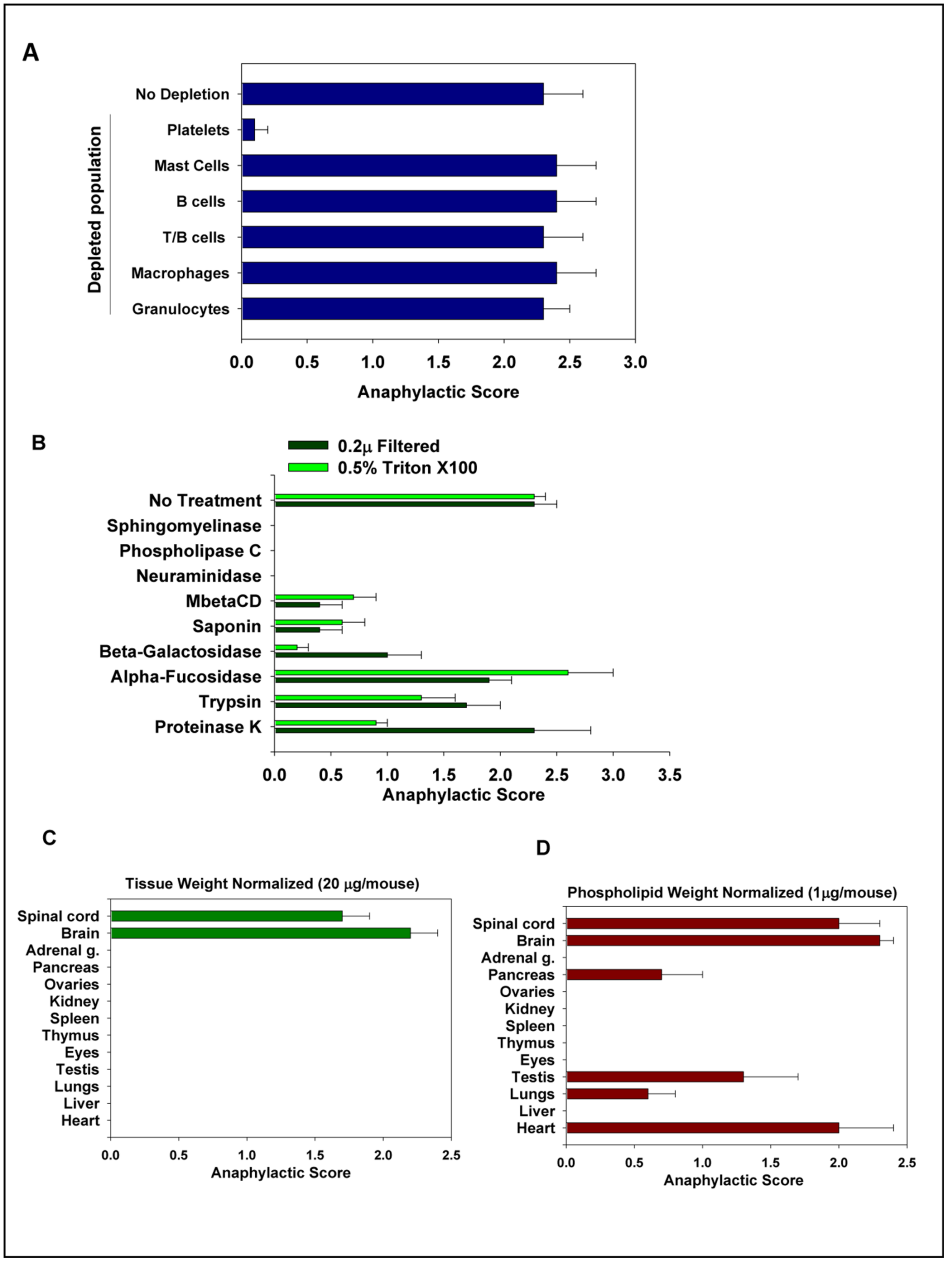
Role of platelets and lipid rafts in the induction of anaphylaxis. (**A**) The lipid rafts were isolated by homogenization of the brain in PBS with 0.5% Triton X-100 and injected i.v. to induce anaphylaxis. The animals were observed for ten minutes as described in *Methods*. The role of platelets and white blood cell subsets were assessed by using an antibody (for depletion of platelets and granulocytes), lyposomes with bisphosphonate clodronate (for depletion of macrophages), or the CD11b-DTR transgenic mice, and genetically deficient animals (mast cells, B cells, T/B cells) as described in *Methods*. (**B**) The effect of enzymatic treatment of brain lipid rafts on their ability to induce anaphylaxis. The brain lipid rafts were obtained by size filtration (0.2 µ filtered) or solubility in 0.5% Triton X-100 (0.5% Triton X-100) as described in *Methods.* The brain lipid rafts were treated with lipases (sphingomyelinase and phospholipase), enzymes that cleave glycopolymers (neuraminidase, β-galactosidase and fucosidase), cholesterol depleting agents (saponin and MβCD) and proteolytic enzymes (trypsin and proteinase K) and injected i.v. for the assessment of anaphylaxis. The non-treated brain lipid rafts were used as a control (no treatment). The animals were observed for ten minutes as described in *Methods*. (**C,D**) The ability of lipid rafts from different organs to cause anaphylaxis. The lipid rafts were isolated from the spinal cord, brain, adrenal gland, pancreas, ovaries, kidney, spleen, thymus, eyes, testis, lungs, liver and heart by the homogenization of tissues in PBS with 0.5% Triton X-100. The amount of i.v. injected lipid rafts was normalized according to the weight of the wet tissue (**c**) or concentration of phospholipids (**d**) as described in *Methods*. The animals were observed for ten minutes as described in *Methods*. In **A–D**, the maximum clinical anaphylaxis score (Mean ± S.E.) of the total number of animals from three separate experiments with 5–6 mice per group in each experiment is shown.l.

In order to study the interactions of blood cells with the structures of the damaged neuronal tissue, we injected the whole brain homogenate intravenously into inbred unmanipulated (non-immunized or naïve) mice. We observed a reaction with the typical features of an anaphylactic shock including the tachycardia, dyspnea and hypothermia (**Figure S1** in **File S1**). To quantitatively assess the symptoms of the observed anaphylactic-like reaction we used an anaphylactic score system: 1 - restless behavior; 2 -loss of consciousness; 3 - dyspnea; 4 - death (see *Methods*). The administration of the brain lipid rafts resulted in 2.3-fold increase in the histamine level in the plasma above the background level, suggesting a quick release of this mediator. The intravenous injection of 1 ml saline substantially ameliorated the symptoms [24] by increasing the volume of the circulating blood and relieving tachycardia, as did the anti-histamine drug pyrilamine by decreasing the anaphylactic score from 1.8±0.2 to 0.8±0.1, further suggesting the involvement of histamine as a mediator of the observed phenomena. The animals that died from the anaphylactic-like reaction did not present necrotic areas in the lungs, a typical sign of thromboembolism. In the subsequent experiments we determined that the shock inducing component was contained in the brain homogenate supernatant centrifuged at low speed (400 g), in agreement with the study by Zibitsker published in 1961 [25]. The size of the component was identified to be between 0.2–0.4 µ and 0.05 µ. The filtered supernatant was sensitive to boiling and treatment with 1% Tween 40, but not to freezing at −20°C or −70^o^, sonication or treatment with 0.5% Triton X-100 (**Table S1** in **File S1**). The resistance to Triton X-100 identified the brain-derived particles which induced the anaphylactic-like reaction as “lipid rafts.” To confirm that the anaphylactic-like reaction was caused by the brain lipid rafts, we isolated them by homogenizing the brain in PBS with 0.5% Triton X-100 and found that the *in vivo* injection of these lipid rafts caused the anaphylactic-like reaction similar to that of 0.2µ filtered supernatants described above. Subsequently, the brain homogenates prepared with 0.5% Triton X-100 were fractionated on a sucrose gradient and analyzed. The anaphilactogenic fractions in the sucrose gradient were particularly enriched in phospholipids and cholesterol and had a low content of the transferin receptor, which are the key features of lipid rafts (**Figure S2** in **File S1**). Regarding the cellular arm of the response we found that the depletion of platelets completely abrogated the anaphylactic-like reaction whereas no effect was observed when macrophages or granulocytes were depleted, or in the mice genetically deficient for mast cells, B cells, or T/B cells (**Figure 1A** and **Table S1** in **File S1**). Finally we performed a differential diagnosis of the clinical and laboratory features of the anaphylactic-like reaction induced by the brain lipid rafts and those of the thromboembolism induced by the i.v. administration of thrombin [26] (**Table S3** in **File S1**; **Video** **S1** and **Video S2)**. In this experiment we demonstrated that the administration of lipid rafts caused anaphylactic-like reaction that is symptomatically different form the thromboembolism induced by the injection of thrombin. Collectively the data suggest that the administration of brain lipid rafts results in the anaphylactic-like reaction mediated by platelets in mice.

### Sialated Gangliosides Induce an Anaphylactic-like Reaction

To understand which components of the lipid rafts are responsible for the platelet mediated anaphylactic-like reaction we enzymatically cleaved lipids, carbohydrates or proteins on the lipid rafts obtained by the size filtration (0.2 µ) and by solubility in 0.5% Triton X-100 (**Figure 1B** **; Table S4** in **File S1**). The treatment of brain lipid rafts with lipases (phospholipase and sphingomyelinase) or neuraminidase (which specifically removes the sialic acid) completely inhibited the anaphylactic response. In addition, the cholesterol-depleting agents MβCD and saponin, and β-galactosidase reduced the anaphylactic-like reaction by ∼80%. Fucosidase (removes fucose) and endoglycosidases (which specifically deglycosylate proteins but not lipids; see **Table S2** in **File S1**) had no effect. The treatment with proteolytic enzymes (trypsin and proteinase K) had a minimal effect on the lipid rafts isolated by the size filtration and decreased the anaphylactic score in the lipid rafts isolated with Triton 0.5% X-100 by ∼50% (**Figure 1B** **; Table S2** in **File S1**). Collectively these findings suggest that the sialated glycosphingolipids (gangliosides) rather than the glycosylated proteins are the primary moieties recognized by the platelets. Indeed, the gangliosides together with other sphingolipids (e.g., sphingomyelin), phospholipids and cholesterol are found within lipid rafts and have a sialated galactose within the glycopolymer [27]. Interestingly, the injection of the “naked” sialated gangliosides (GM1, GD3 and GT1b) did not result in an anaphylactic-like reaction. In addition to that, removing cholesterol (using saponin or MβCD) that is necessary for the structural stability of lipid rafts decreased the ability of the rafts to cause the anaphylaxis by ∼80%. Taken together these results indicate that platelets recognize gangliosides only within the intact structure of lipid rafts.

Gangliosides are present in many tissues, but the most abundant source is the brain [28]. We isolated lipid rafts from different organs and normalized the amount of injected rafts to the initial wet weight of the tissue. As shown in **Figure 1C**, only the lipid rafts from the brain and spinal cord triggered the anaphylactic-like reaction *in-vivo*. Since the lipid rafts were isolated from the same amount of tissue, we hypothesized that the amount of lipid rafts in organs varies. Indeed, when we measured the concentration of phospholipids, we found out that the internal organs had less lipid rafts compared to the brain (**Table S4** in **File S1**). When the amount of the injected rafts was normalized to the concentration of phospholipids, we found that the lipid rafts from the testis, pancreas, lungs and heart also evoked the anaphylactic-like reaction (**Figure 1D** **, Table S4** in **File S1**). Of interest is that the testis and pancreas are considered to be the “barrier” organs similar to the CNS, whereas the heart is prone to ischemia, an attribute of inflammation and injury, similar to a stroke in the brain. In summary, these data suggest that: 1) the brain and spinal cord have the highest concentration of the lipid rafts that are recognized by the platelets, and 2) the organized structures of sialated gangliosides within the lipid rafts are required for the recognition of these structures by the platelets leading to anaphylaxis.

### The Lipid Rafts on Astrocytes and Neurons Induce an Anaphylactic-like Reaction

**Figure 2:**
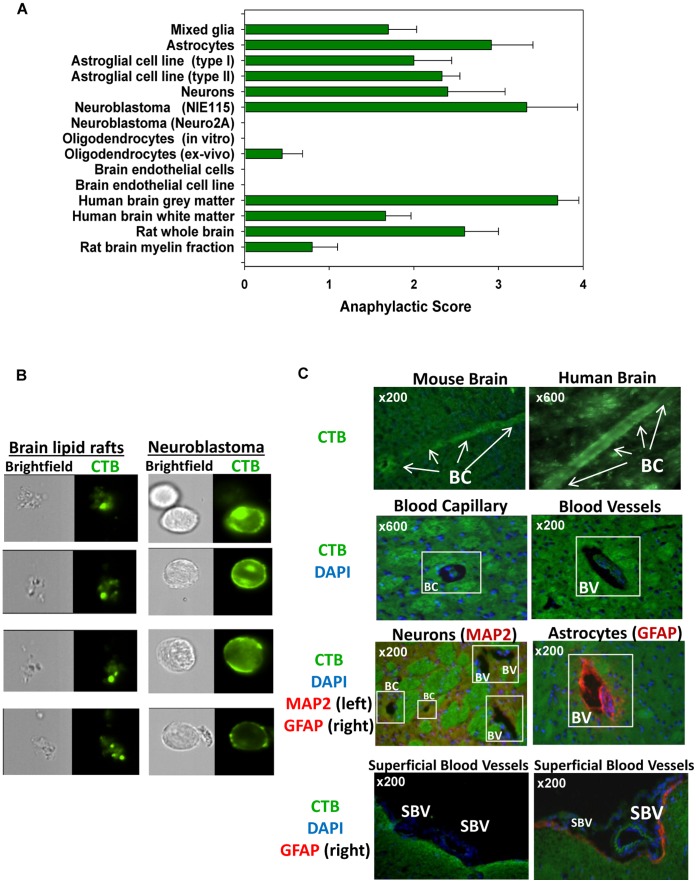
Origin and localization of brain lipid rafts. (**A**) The lipid rafts were isolated from the primary cultures, cell lines, human brain gray and white matter, or rat whole brain and rat brain myelin fractions. The lipid rafts were injected i.v. and anaphylaxis was scored (see **Figure 1**). (**B**) The brain homogenates and neuronal line cells lipid rafts were stained for a GM1 marker of lipid rafts (CTB) and visualized by Imaging Cytometry (green). (**C**) The sections of the mouse (left) and human (right) brains stained with CTB (top two images, green). In the middle four images, the mouse brain sections stained with CTB show the lipid rafts around the brain capillaries (left) and blood vessels (right); same with double-staining for the neuronal (MAP2; left) and astroglial (GFAP; right) (both red) markers. Two bottom images depict areas around the brain blood vessels under pia matter (superficial blood vessels) single stained with CTB (left, green) or double stained with CTB/GFAP (right; CTB in green, GFAP in red). DAPI staining for the cell nuclei is shown in blue. *Abbreviations:* BC – brain capillary, BV-blood vessel, SBV-superficial blood vessel.

To find the cellular source of the lipid rafts responsible for the platelet-mediated anaphylactic-like reaction, we tested the cell lines and primary cells from the CNS. The lipid rafts from the cells of the neuronal and astroglial origin caused an anaphylactic-like reaction, whereas the rafts from the cells of oligodendroglial and endothelial origin did not (**Figure 2A**). Similar to the brain lipid rafts described above, the enzymatic analysis demonstrated that the lipid rafts from astroglial cells induced the anaphylactic-like reaction in mice in a sialoganglioside-dependent fashion. (**Figure S3** in **File S1**). Of note, the lipid rafts from the neuroblasoma cell line NIE115 were also anaphylactogenic, whereas from the line Neuro2A were not. Neuro2A is deficient in sialated gangliosides including GD3 and GQ [29] which is consistent with our findings that the sialated gangliosides are recognized by the platelets during the induction of the anaphylactic-like reaction. To compare the lipid rafts from the species other than mice, we injected lipid rafts from human, rat, and fish brains into mice. The anaphylaxis was observed with human and rat, but not fish brains. We then isolated the lipid rafts separately from the white and gray matter of the human autopsy brain samples. The gray matter (enriched with astrocytes and neurons) lipid rafts had more than a 2-fold higher effect when compared with the white matter (enriched with oligodendrocytes) rafts **(** **Figure 2A**). Similarly, the lipid rafts isolated on a sucrose gradient from the rat brain myelin fraction (enriched in oligodendrocytes and normalized by the raft content were 3-fold less anaphylactogenic compared to the lipid rafts isolated from the whole rat brain **(** **Figure 2A**). In addition, we found that the rat cortical neurons and astrocytes caused anaphylaxis, whereas the rat oligodenodrocytes did not (not shown).

### The Lipid Rafts of Astroglial and Neuronal Cells Carry GM1, GD3, GT1b and GQ

It has been reported that the subunit B of Cholera toxin (CTB) binds GM1 ganglioside, the ubiquitous marker of lipid rafts [30]. We visualized lipid rafts with fluorescent CTB using the imaging cytometry and found that the brain lipid rafts had round compact areas ∼0.5 µ or less in diameter with bright staining for CTB (**Figure 2B**, Brain lipid rafts). These lipid rafts were also present on the anaphylactogenic neuroblastoma cell line NIE115 (**Figure 2B**, Neuroblastoma). We then examined the expression of several sialated gangliosides GM1, GM2, GD3, GD1b, GT1b and GQ on the following: the brain lipid rafts, neurons, neuroblastoma cell line, astrocytes and astroglial cell line (**Figure S4** in **File S1**). All the lipid rafts (both from the brain and the cells of the neuronal/astroglial origin) we tested expressed the following four gangliosides: GM1, GD3, GT1b and GQ.

### Lipid Rafts are Co-localized with Perivascular Astrocytes and Neurons

We investigated the anatomic location of lipid rafts in the mouse and human brains by staining frozen sections with fluorescent CTB. Although lipid rafts could be found throughout the brain, the brightest staining occurred around the long pipe-like structures of the brain capillaries (**Figure 2C**, top images). Using single staining for GM1 on the lipid rafts (CTB), and double staining for the lipid rafts (CTB) and neurons (MAP2), or astrocytes (GFAP), we found GM1 positive astrocytes and neurons surrounding the brain capillaries and blood vessels (**Figure 2C**, middle images). We also found areas of bright staining for GM1 on the astrocytes underlying the brain subpial (superficial) blood vessels (**Figure 2C**, bottom images). To confirm the presence of lipid rafts on the neuronal cells ex-vivo, we used Thy1-YFP reporter mice that have neuronal bodies and axons (including neuronal endings) genetically labeled with YFP [31]. The YFP positive fluorescent neuronal axons were also GM1 positive in the brain areas close to the blood vessels and capillaries, known as neurovascular units (**Figure S5** in **File S1**). In contrast, we found no co-localization of GM1 staining with the staining for basal membranes, endothelial cells or pericytes either in the mouse or human brains (**Figure S6** in **File S1**). Thus, lipid rafts are preferentially detected on the astrocytes and neurons of neurovascular units of the intact brain.

### Platelets Interact with Natural and Model Lipid Rafts

**Figure 3:**
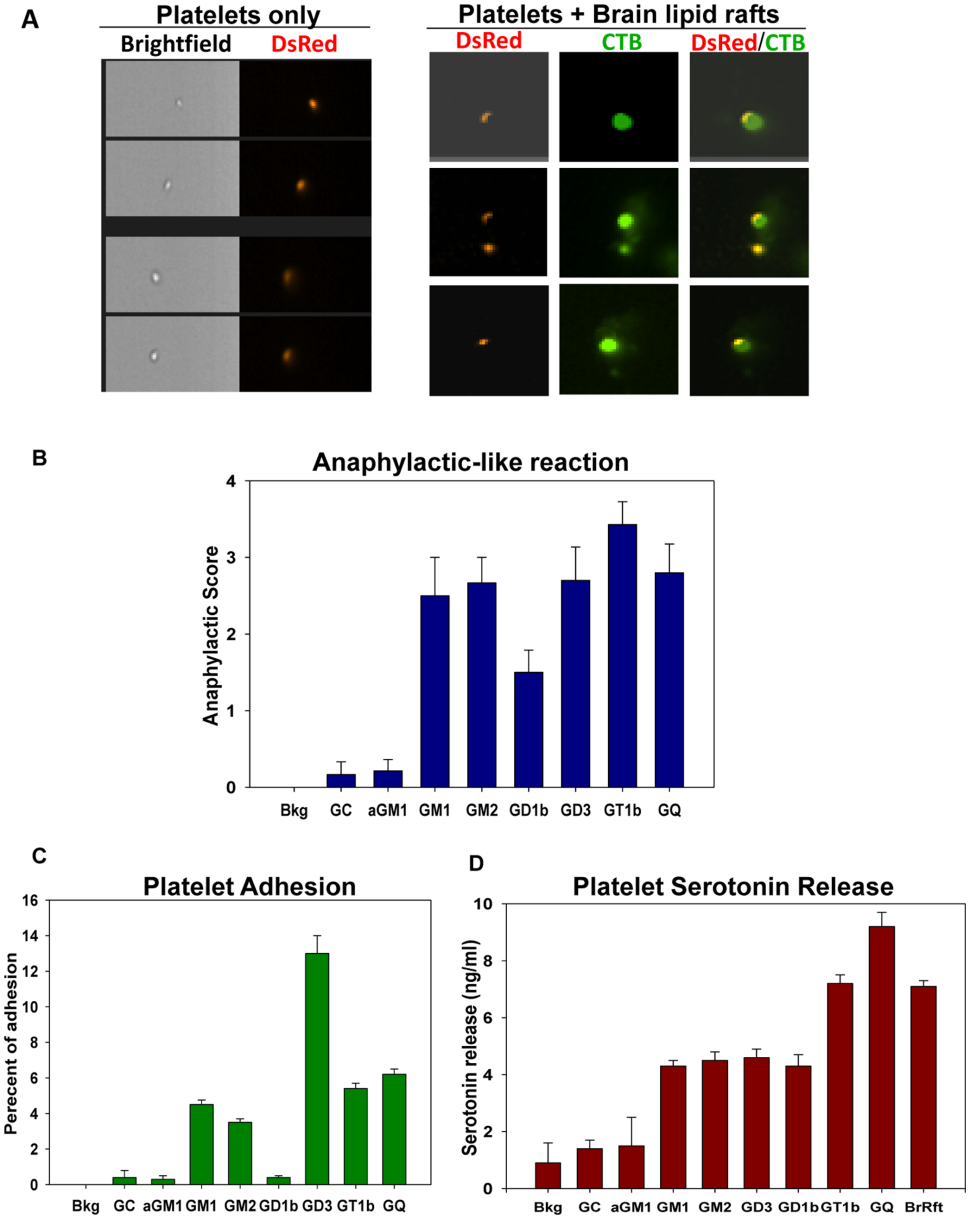
Unique composition of lipid rafts triggers platelets degranulation. (**A**) The platelets were isolated from the DsRed transgenic mice, incubated with CTB-prestained brain lipid rafts for 3 minutes, fixed, washed and analyzed by Imaging Cytometry. Left, the bright field images and platelets fluorescence is shown (red). Right, the platelets (red) interaction with brain lipid rafts (CTB, green) is shown. (**B**) The induction of anaphylaxis by model lipid rafts *in vivo*. The model lipid rafts containing representative gangliosides (asyaloGM1, GM1, GM2, GD3, GD1b, GT1b, GQ), galactosylcerebroside (GC) or phosphatidylcholin (Bkg) were prepared and injected i.v. into the mice as described in *Methods*. The maximum clinical anaphylaxis score (Mean ± S.E.) of the total number of animals from three experiments with 4–5 mice per group in each experiment is shown. (**C**) The platelet adhesion to gangliosides *in vitro*. AsialoGM1, GM1, GM2, GD3, GD1b, GT1b, GQ1b gangliosides, galactosylcerebroside (GC) or phosphatidylcholin (Bkg) were adsorbed to flat-bottom 96-well plates and the adhesion of platelets to the ligands was measured. (**D**) The platelets serotonin release induced by the model lipid rafts *in vitro.* The model lipid rafts were incubated with platelets and the concentration of serotonin in the platelet-free supernatant was assessed. The brain lipid rafts (BrRft) served as a positive control. In **c–d**, the mean ± S.E. of triplicate is shown. The data is representative of two separate experiments.

Since the intravenous injection of the brain lipid rafts led to the anaphylaxis mediated by platelets and required sialated gangliosides on the lipid rafts (**Figure 1**), we hypothesized that platelets first adhere to lipid rafts and then degranulate to induce an anaphylactic-like reaction. Our hypothesis was supported by the reports of a massive platelets degranulation caused by the intravenous administration of a radiographic contrast media in the humans [32] or by lyophilized whole bacterial cells in mice causing an anaphylactic shock [33]. To test whether platelets interact with lipid rafts *in vitro*, we mixed the platelets isolated from DsRed transgenic mice that ubiquitously express the red fluorescent protein under the actin promoter (**Figure 3A**, Platelets only) with the brain lipid rafts from naïve inbred mice (pre-stained with fluorescent CTB) and after 3 minutes the samples were fixed and analyzed by the imaging cytometry. We found that the platelets (red) adhered to the lipid rafts (green) (**Figure 3A**, Platelets+Brain lipid rafts). The co-incubation of mouse platelets with either the mouse or human brain lipid rafts *in vitro* resulted in the release (7–10 fold upregulation) of serotonin (**Figure S7** in **File S1**), PF4/CXCL4 and the anaphyloctogenic mediator histamine (not shown), a hallmark of the platelet degranulation. In a reverse experiment we demonstrated that the incubation of human platelets with human or mouse lipid rafts also resulted in a serotonin release (**Figure S7** in **File S1**) suggesting that a similar mechanism of platelet degranulation in response to the presence of lipid rafts takes place in humans. In addition, we found that the interaction of human or mouse platelets with the brain lipid rafts *in vitro* resulted in the platelet shape change and aggregation (**Figure S8** in **File S1**). We also observed the upregulation of the activated form of αIIbβ3 integrin on the platelets (**Figure S8** in **File S1**), Ca^2+^ influx, and secretion of IL-1α (**Figure S9** in **File S1**). This suggests that platelets become activated and degranulate upon the interaction with lipid rafts.

To understand the role of gangliosides in the lipid raft-induced platelet adhesion and degranulation we created artificial lipid rafts with various integrated gangliosides and then tested the ability of these lipid rafts to induce anaphylaxis. We found that the i.v. injection of the model rafts with sialated gangliosides resulted in an anaphylactic-like reaction, with ganglioside GT1b eliciting the strongest and GD1b the weakest effect. At the same time, the lipid rafts without gangliosides (Bkg) or containing an asialated GM1 ganglioside (aGM1) or galactocerebroside (GC) did not induce anaphylaxis (**Figure 3B**).

The role of ganglioside GD3 has been reported in the platelet adhesion [34]. We tested the gangliosides described above to find that platelets primarily adhered to GD3 with some binding to GT1b and GQ1b, as well as to GM1 and GM2 (**Figure 3C**). Next we assessed the ability of artificial lipid rafts with selected gangliosides to cause the platelet degranulation *in vitro* as determined by the serotonin release. GT1b and GQ1b caused the most prominent release of serotonin at a level similar to the natural brain lipid rafts. The model lipid rafts without gangliosides (Bkg) or with asialated GM1 (aGM1) or galactocerebroside (GC) did not cause a serotonin release above the background levels (**Figure 3D**).

### Platelets Receptors Recognize Sialated Gangliosides

Since platelets recognize sialated gangliosides within the lipid rafts, we carried out experiments to identify the receptors responsible for this interaction. In the first series of the experiments we blocked the receptors with antibodies or used the mice genetically deficient for the known platelet receptors and other factors (including integrins, FcR gamma chain, scavenger receptors; see **Table S5** in **File S1**), but found that none of these molecules were responsible for the anaphylactic-like reaction. Our results (above) indicated the involvement of the receptors that specifically recognize the sialic acid on charbohydrate polymers. Indeed, two classes of receptors are known to recognize the sialated carbohydrate structures, including gangliosides: lectins and siglecs [35]. Platelets are known to express P-selectin (CD62P) [10]; the expression of siglecs on the platelets has not been examined. In addition, the adhesion molecules of the immunoglobulin family can potentially bind sialated carbohydrates [36]. We found that mouse platelets express two activating siglec receptors, Siglec-H and Siglec-15 (**Figure S10a** in **File S1**). Of the examined adhesion molecules, the mouse platelets only expressed ALCAM/CD166 (**Figure S10a** in **File S1**) as determined by FACS. We confirmed the expression of ALCAM on the mouse and human platelets by Western blot (**Figure S10b** in **File S1**). We also examined the expression of adhesion molecules in the human platelets by the flow cytometry and found that the human platelets also expressed ALCAM (**Figure S10c** in **File S1**). We detected the baseline level of expression of P-selectin on *ex-vivo* isolated platelets in the platelet rich plasma of mice and human (**Figure S10a,c** in **File S1**), which could be explained by a partial activation of platelets during the isolation and antibody staining; however P-selectin was substantially unregulated when the platelets were activated *in vitro* (not shown).

**Figure 4:**
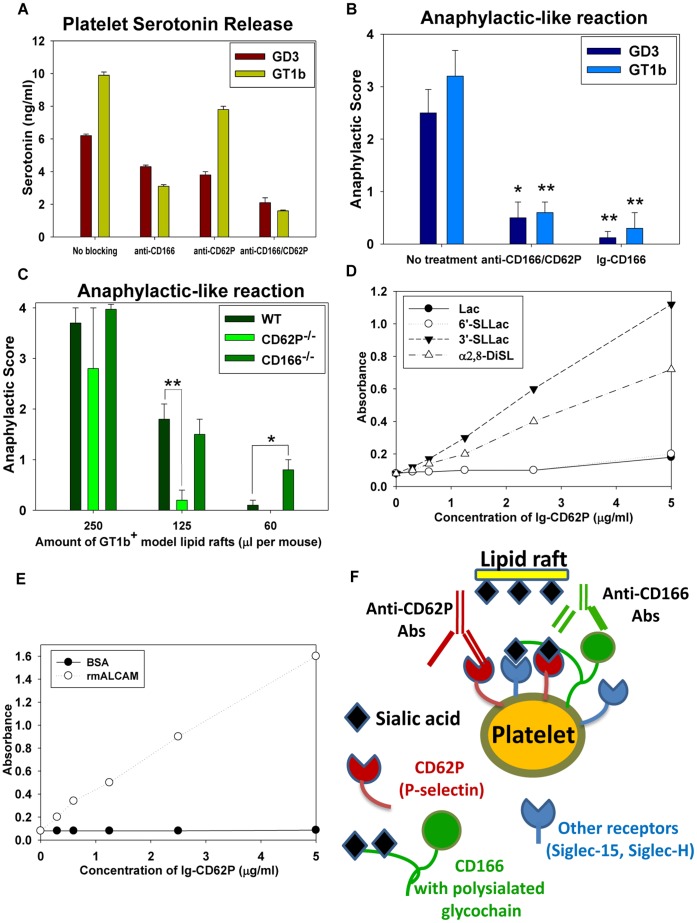
Lipid rafts engage platelet receptors specific to sialated gangliosides. (**A**) The effect of anti-CD62P and anti-CD166 mAbs on the platelet serotonin release in response to the model lipid rafts containing GD3 and GT1b *in vitro*. The serotonin release was measured (see **Figure 3**) in the presence of anti-CD62P and anti-CD166 mAbs. (**B**) The effect of anti-CD62P and anti-CD166 mAbs or the CD166-Fc fusion protein on the platelet degranulation iduced by the model lipid rafts *in vivo*. Anti-CD62P and anti-CD166 mAbs were injected i.v., and the model lipid rafts containing GD3 or GT1b gangliosides were injected 30 minutes later. The model lipid rafts containing GD3 or GT1b gangliosides were pre-treated with the CD166-Fc protein and injected i.v. into naïve mice. All mice were then injected i.v. with the lipid rafts as described in *Methods*. The maximum clinical anaphylactic score (Mean ± S.E.) of the total number of animals from three experiments with four mice per group is shown (*, p<0.05; **, p<0.01; when compared to non-treated animals). (**C**) The lipid rafts-induce anaphylaxis in wild-type (WT), CD62P (P-Selectin)- and CD166 (ALCAM)-deficient mice. The model lipid rafts containing GT1b ganglioside were injected i.v. into naïve mice at 250, 125 or 60 µl per mouse. The maximum clinical anaphylaxis score (Mean ± S.E.) of the total number of animals from the three experiments with 4 mice per group in each experiment is shown (*, p<0.05; **, p<0.01; when compared to wild-type animals). (**D**) Binding of CD62P-Fc fusion protein to lactose, 6′-sialyllactose, 3′-sialyllactose and α2,8-disialic glycopolymers adsorbed onto 96 well plates. (**E**) Binding of CD62P-Fc fusion protein to recombinant ALCAM or BSA (control) adsorbed onto 96 well plates. (**F**) Schematics of multiple receptors (CD62P, ALCAM, Siglec-H and Siglec-15) on the platelets and sialated gangliosides on brain lipid rafts interaction. In addition to the interaction of sialic acid on gangliosides with CD62P and Siglecs, ALCAM has mono and poly- sialated glycoepitopes that can be recognized by CD62P and Siglec-H/Siglec-15. The antibodies to ALCAM prevent binding of masked CD62P and other siglec receptors on the platelets to the sialated gangliosides on the lipid rafts. Thus, the anti-ALCAM and anti-CD62P antibodies disrupt an intricate network of the lipid raft-sensing receptors on the platelets surface.

In the second series of the experiments we tested whether an antibody to CD62P, Siglec-H, Siglec-15 or CD166 could block the ganglioside GT1b or GD3-mediated serotonin release by the platelets. The separate anti-CD62P and anti-CD166 treatment partially decreased the serotonin release and the simultaneous addition of the Abs showed the most inhibition (**Figure 4A**). The anti-Siglec-15 and anti-Siglec-H antibodies did not affect the serotonin release in our assays, suggesting that either these receptors do not play a role in recognizing the sialated gangliosides or the antibodies failed to block the interaction. Finally we tested whether the classic platelet receptors GPIb/CD42 play a role in the recognition of the brain lipid rafts and found that the blocking antibodies to CD42 failed to block the serotonin release *in vitro* induced by the natural and model lipid rafts (not shown).

*In vivo,* the systemic (i.v.) injection of both anti-CD62P and anti-CD166 Abs substantially ameliorated the anaphylaxis induced by the model lipid rafts containing GD3 or GT1b (**Figure 4B**). Also, the anaphylaxis was inhibited by the pretreatment of the GD3 or GT1b-containing rafts with a soluble CD166-Fc fusion protein to compete with the cell-surface CD166 (**Figure 4B**). Using genetically modified mice we demonstrated that the CD62P deficient mice had a lower anaphylactic score when treated with lipid rafts compared to the wild type B6 mice (**Figure 4B**, CD62P^−/−^). On the other hand, the CD166 deficient mice had similar or higher anaphylactic scores (**Figure 4C**, CD166^−/−^). Those were unexpected results, since antibodies to CD166 decreased the anaphylaxis (**Figure 4B**). We hypothesized that CD62P binds sialated glycoepitopes on gangliosides directly, whereas ALCAM interacts with CD62P to inactivate or mask it as was reported for several siglecs (e.g. CD45 binds and inactivates Siglec-2) [37]. ALCAM has mono- and di- sialated glycoepitopes in the structure that closely mimics gangliosides [29] and these regions of ALCAM could potentially bind CD62P and prevent its interaction with the gangliosides in the lipid rafts. In our experiments we showed that the CD62P-Fc fusion protein did bind the mono- and di- sialated glycoepitopes (3′-sialyllactose and α2,8-disialic acid) that are present on the gangliosides (**Figure 4D**), whereas ALCAM did not (not shown). We also found that ALCAM could bind CD62P-Fc (**Figure 4E**), suggesting that ALCAM could interfere with the activation of platelets via binding to the cell-surface CD62P. This explains why the antibodies to CD166 and CD62P blocked the platelet-raft interactions (**Figure 4F**). Since P-selectin is expressed on the surface of the activated platelets, we believe other receptors that recognize the sialic acid (e.g. Siglec-H, Siglec-15) are involved in the initiation of the lipid rafts recognition by the platelets with the outcome of the platelets activation, expression of P-selectin and development of anaphylaxis (**Figure 4F**).

### Platelets Interact with Lipid Rafts in the CNS during Neuroinflammation

**Figure 5:**
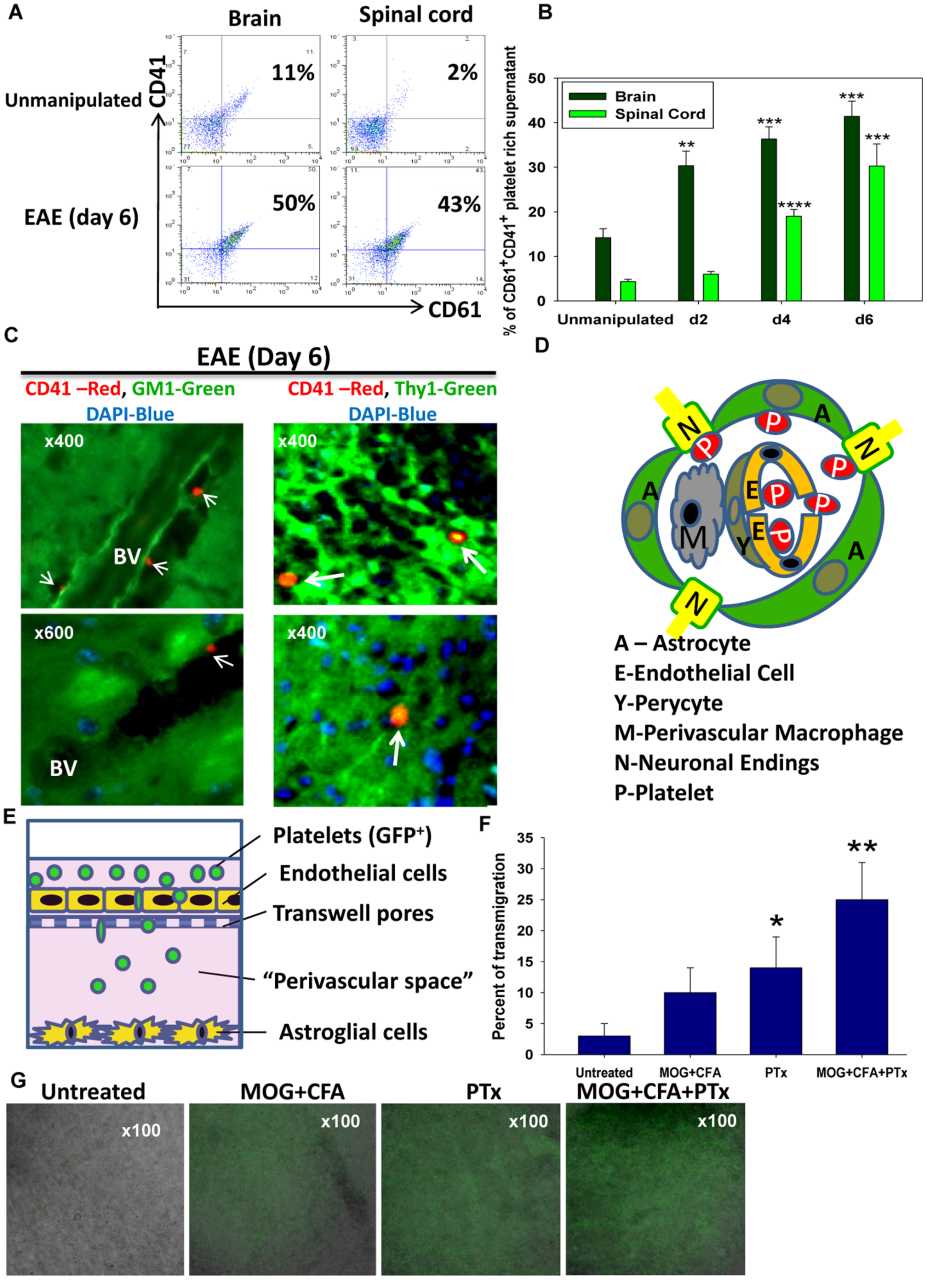
Platelets penetrate the blood-brain barrier (BBB) and accumulate in the lipid rafts-rich areas in the model of neuroinflammation. (**A–C**) The platelets isolated from the CNS of healthy mice or mice with EAE were analyzed by flow cytometry. The platelet-rich supernatants were prepared from the CNS homogenates, the platelets were stained for CD41 and CD61 (**A**) The percentages of CD41^+^CD61^+^ platelets are depicted in the upper right quadrants of the contour plots. (**B**) The increase of platelet numbers in the brain and spinal cord in the early stages of EAE (Mean ± S.E, five animals per group; **, p<0.01; ***, p<0.001; ****, p<0.0001; when compared to the unmanipulated mice). (**C**) The immunofluorescent analysis of the platelet localization within the CNS of WT B6 (**C**) or the Thy1-YFP reporter mice (**D**) with EAE on day 6. The CNS sections were double stained with CTB (green) and anti-CD41 mAbs (red) in (c, left panel) or single stained with anti-CD41 (red) in (c, right panel). The CD41^+^ platelets are indicated by the arrows. (**D**) The schematic model of platelets topographic disposition in the perivascular space and their interaction with lipid rafts on astrocytes and neurons as a result of the increased CNS blood vessel permeability. (**E–F**) The model of the BBB with a “perivascular space” between the monolayer of brain endothelial cells in the top chamber and the astroglial cells in the bottom chamber of the Transwell system (**E**). The platelets isolated from the ACTB-GFP transgenic mice were added to the top chamber together with MOG/CFA and/or PTx. The percentage of the transmigrated GFP^+^ platelets is shown in (**F**). The combined bright field (astroglial cells, grey) and fluorescent images (GFP^+^ platelets, green) of the bottom chambers under specified conditions are shown in (**G**).

We next investigated the platelet-lipid raft interaction in the CNS during EAE, a classic model of inflammation in the CNS. We found that CD41^+^CD61^+^ platelets were present in the normal brain (14% of the brain homogenate platelet-rich supernatant), and the spinal cord (5%). In the initiation phase of EAE, on day 2 for the brain and day 4 for the spinal cord, there was a substantial increase in the platelets counts (**Figure 5A,B**) and platelets activated phenotype as determined by the CD62P upregulation (not shown). On the frozen brain sections the CD41^+^ platelets (red staining) were detected in the perivascular spaces adjacent to the GM1^+^ brain lipid rafts (green staining) on day 6 after the EAE induction (**Figure 5C**). We showed that the neuronal cells in the Thy1-YFP reporter mice had lipid rafts (**Figure S5** in **File S1**). Accordingly, on day 6 of EAE in the Thy1-YFP reporter mice [31] we registered the platelets (often in aggregates) in a close proximity to the YFP^+^ neuronal bodies and axons (**Figure 5D** **; Figure S11** in **File S1**). The schematics of the platelets interaction with the lipid rafts on astrocytes and neuronal endings of the perivascular areas in the early stages of EAE is shown in **Figure 5E**. In the more advanced stages of EAE (the peak of the disease), platelet aggregates and single platelets were also located in the CNS parenchyma close to the infiltrating CD11b^+^ macrophages as shown in **Figure S12** in **File S1**.

To interact with lipid rafts on the surface of astrocytes and neurons platelets must transmigrate into the CNS through the tight junctions of the brain blood-vessel endothelial cells. It is reported that platelets acquire the ability to move through the activated endothelial cells during inflammation [38]. In the EAE model, mice were immunized with MOG/CFA, and *Pertussis toxin* (PTx) was administered on day 0 and 2 post-immunization. It has been shown that PTx increases the endothelium permeability both *in vivo* [39], [40] and *in vitro* [41], [42]. We found that both MOG/CFA and PTx affect the BBB permeability on day 3 of EAE as determined by the penetration of FITC-dextran from the blood into the brain and spinal cord (not shown). Next we examined whether the activation of endothelial cells with MOG/CFA and/or PTx would increase the platelets transmigration in *vitro*. We created a model of the BBB by culturing astroglial cells on the bottom of a transwell lower chamber, and endothelial cells on the top side of the upper chamber filter as previously described [41], [43] (**Figure 5E**). Both MOG/CFA and PTx dramatically increased the transmigration of platelets through the confluent monolayer of endothelial cells towards the astroglial cells stratum (**Figure 5F,G**). The percentage of transmigration into the lower chamber is shown in **Figure 5F**. In **Figure 5G** the relative quantity of the GFP positive platelets (shown in green) on the astroglial layer (shown in grey) in the lower chamber could be also estimated by eye as the intensity of the green color and the area that is covered by the GFP^+^ platelets. Next, we examined the platelet reaction to the brain lipid rafts in a EAE model. The mice with EAE had an almost 2-fold higher anaphylaxis score after i.v. injection of brain lipid rafts compared to the healthy controls (**Figure S13a** in **File S1**). We hypothesized that this was related to the ongoing processes of degranulation of platelets in the CNS during EAE. It was reported that platelets accumulate and degranulate in the lungs in the models of airway inflammation [44], while the CD41^+^ microparticles and PF4/CXCL4 serve as markers of that degranulation [8], [45]. There was an increase of CD41^+^ particles in the plasma of mice with EAE (**Figure S13b** in **File S1**). Moreover, mice with EAE had 25-fold higher levels of PF4 in the serum compared to the healthy mice, and similar to the brain lipid rafts-treated mice (**Figure S13c** in **File S1**). Thus, during neuroinflammation, platelets interact with brain lipid rafts and are highly sensitive to the degranulation in the periphery.

### Recognition of Brain Lipid Rafts by Platelets Plays an Important Role in the Development of Neuroinflammation

**Figure 6:**
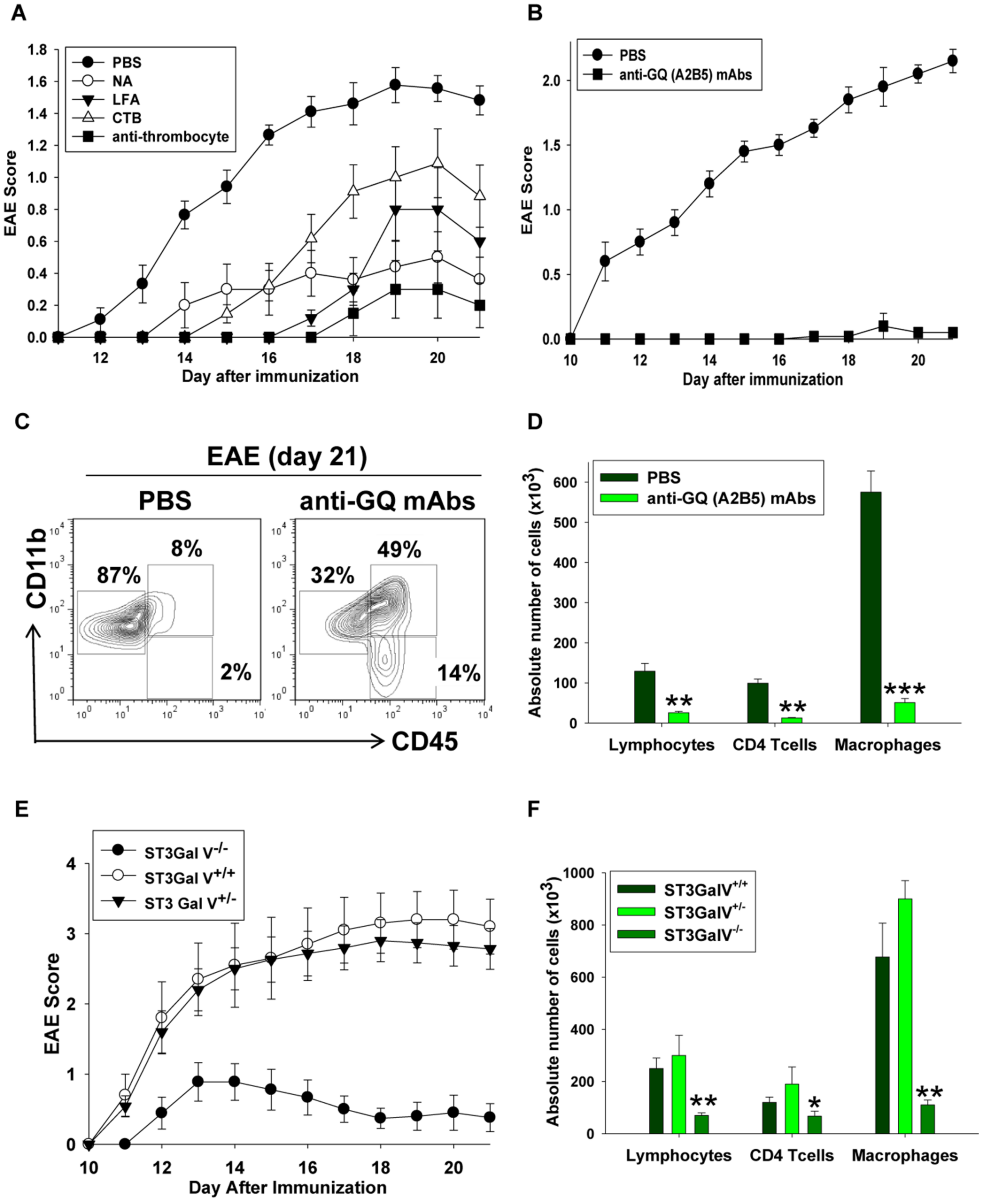
Effects of the agents that affect the platelet-lipid raft interactions in the development of EAE. (**A,B**) EAE in mice treated with anti-thrombocyte serum (anti-thromocyte), beta subunit of cholera toxin (CTB), neuroaminidase (NA), and *Limax flavus* agglutinin (LFA) (**A**) or anti-GQ (A2B5) antibodies (**B**). EAE was induced by the immunization of B6 with MOG/CFA as described in *Methods*. *Pertussis toxin* (PTx) was administered i.p. on day 0 and day 2 post-immunization. The anti-thrombocyte serum was injected on day 0, 2, 4 and 8 post-immunization, and CTB, NA and LFA were injected on days 0, 2, 4, 6 and 8 post-immunization (**A**). The anti-GQ antibodies were injected on days 0, 2, 4, 8, 10, 12 and 14 post-immunization (**B**). The mice were monitored daily and the EAE score was assessed as described in *Methods.* Mean ± S.E. of the total number of animals from the three separate experiments with groups of 7–10 mice in each experiment is shown. (**C,D**) The flow cytometry analysis of the CNS infiltrating cells isolated from the mice treated with anti-GQ antibodies. The mononuclear cells were isolated from the CNS of the mice treated with PBS or anti-GQ antibodies on day 21 after EAE induction, stained for CD11b and CD45 and analyzed by flow cytometry as described in *Methods*. The staining for CD11b (y-axis) and CD45 (x-axis) of the CNS mononuclear cells is shown. The percentages of populations of resting CD11b^+^CD45^low^ microglia (left gates), CD11b^+^CD45^hi^ macrophages (upper right gates) and CD11b^−^CD45^hi^ lymphocytes (lower right gates) are shown in (**C**). The quantification of the absolute number of macrophages, lymphocytes and CD4 T cells in the CNS is shown in (**D**). The absolute numbers of CD11b^−^CD45^hi^ lymphocytes, CD3^+^CD4^+^ T cells and CD11b^+^CD45^hi^ macrophages were calculated by multiplying the total cell count obtained using a hemocytometer by the percentage of these cells determined by flow cytometry. Mean ± S.E. of the group of five animals is shown. (**E,F**) The EAE disease course (**E**) and the quantification of the CNS immune cell infiltrate (**F**) in the groups of ST3Gal-V^−/−^, ST3Gal-V^+/−^ and ST3Gal-V^+/+^ mice. The EAE was induced and cellular infiltration was analyzed as in (**A**) and (**D**), respectively. The mean ± S.E. of the total number of animals from the three separate experiments with the groups of 7–10 mice in each experiment is shown.

We then asked whether the recognition of lipid rafts by platelets played a role in the EAE progression. It was recently reported by Langer *et al.* that platelets participate in the EAE pathogenesis by recognizing integrins [19]; we hypothesized that the platelet-rafts interactions also substantially contributed to the disease. To test this hypothesis, we depleted the platelets or targeted ligands on the lipid rafts to interfere with the platelet recognition of lipid rafts. We depleted the platelets by injecting the anti-thrombocyte serum on days 0, 2, 4 and 8. In addition, we treated animals with neuroaminidase (NA) from *C. Perfringers,* which removes the terminal sialic acid from glycoproteins and glycolipids by cleaving the α2–3, α2–6, and α2–8 linkages, and also decreases the platelet numbers. Finally, we used a LFA protein which specifically binds the sialic acid [36], [46] and CTB which binds GM1 ganglioside in the lipid rafts [30], [36] to prevent the interaction of the platelets with lipid rafts. As shown in **Figure 6A**, we found that each of these treatments ameliorated the EAE symptoms, demonstrating that the platelet-lipid rafts interactions could play an important role in EAE. However NA, CTB and LFA are considered to be “broad spectrum agents” that affect not only the platelet-raft interactions but also other cells since the sialated glycoepitopes are present on many cell types on both glycoproteins and glycolipids. To exclude the non-specific effects of broad spectrum agents on EAE, we used other approaches to specifically block the interaction of platelets with lipid rafts on the astrocytes and neurons. We used the A2B5 antibodies that bind ganglioside GQ which is expressed specifically on the neuronal and astroglial cells [47] and abundantly expressed in the brain lipid rafts (**Figure S4** in **File S1**). We found that the treatment with an anti-GQ antibody substantially ameliorated EAE (**Figure 6B**), and decreased the infiltration of the CD4 T cells, lymphocytes and macrophages in the CNS (**Figure 6C,D**). On the other hand, the anti-GQ antibodies did not affect either the priming or effector functions of the encephalitogenic T cells (**Figure S14** in **File S1**), suggesting that these antibodies did not have a non-specific direct effect on immune cells. To confirm the data obtained with broad spectrum agents (NA, CTB, LFA) and specific anti-GQ antibodies, which block the platelet-rafts interactions, we used the mice genetically deficient for sialyltranferase ST3Gal-V (GM3 Synthase; gene: *Siat9*). These mice lack brain-specific gangliosides such as GT1b and GQ; however the glicosylation of proteins remains intact [48]. We found that the clinical manifestation of EAE was substantially ameliorated in ST3Gal-V^−/−^ when compared to ST3Gal-V^+/−^ and ST3Gal-V^+/+^ control groups (**Figure 6E**). In addition, the CNS inflammation was significantly reduced as determined by the infiltration of lymphocytes, CD4 T cells and macrophages on d21 after the EAE induction (**Figure 6F**). Thus our study demonstrates that preventing the interaction between platelets and brain-derived lipid rafts in the CNS by various methods substantially ameliorated EAE.

### Interaction of Platelets with Lipid Rafts in the CNS and the Periphery Results in Induction of EAE in the Absence of *Pertussis toxin*

To induce EAE, *Pertussis toxin* (PTx) is required in our model. Although PTx may have multiple effects on immune cells, one of the main mechanisms of its action is “opening” the BBB [49] and, as we suggested, the facilitation of the platelet migration into the CNS and their interaction with lipid rafts *in situ*. We investigated whether the massive degranulation of platelets induced by lipid rafts in the periphery or in the brain contributed to the EAE induction in the absence of PTx. We immunized mice with MOG and induced a systemic degranulation of the platelets by injecting the brain lipid rafts i.v. on day 0 (**Figure S15a** in **File S1**). In a separate experiment we induced the degranulation of platelets by lipid rafts only in the brain by injecting purified platelets intracranially (**Figure S15b** in **File S1**). We found that in both cases EAE was induced in the absence of PTx. Thus we believe that the injection of lipid rafts i.v. or platelets i.c. results in systemic or local inflammatory responses and exacerbation of EAE as a consequence of the interactions of platelets with brain gangliosides in lipid rafts and the production of proinflammatory mediators (e.g. PF4, IL-1α) by the platelets.

### Interactions of Platelets with Lipid Rafts Result in Microglia Activation, Transient Infiltration of Macrophages and Lymphocytes in the CNS and Granulocytosis in the Periphery

Based on the previous results we hypothesized that the interaction of platelets with lipid rafts after a systemic administration of lipid rafts or during EAE resulted in the platelet degranulation and the release of pro-inflammatory mediators, which in turn contributed to the activation of the CNS-resident innate immune cells and influx of leukocytes from the periphery. To assess this we examined immune cells in the CNS and spleen after the systemic administration of lipid rafts. We found that 10 minutes after the i.v. injection of brain lipid rafts, microglia upregulated MHC class II and the numbers of macrophages were increased both in the CNS and the spleen (**Figure S16a,c,d** in **File S1**). Beginning at one hour, lymphocytes were also detected in the CNS (**Figure S16e** in **File S1**). The percentage of the activated microglia and the numbers of infiltrating lymphocytes and macrophages returned to normal in the CNS by 48 hrs (**Figure 16a,c,e** in **File S1**); the numbers of granulocytes remained increased in the spleen after 48 hrs (**Figure 16b** in **File S1)**. Taken together, these data suggest that the interaction of platelets with lipid rafts results in the activation of cells of immune system both in the periphery and in the CNS.

## Discussion

This study uncovers new ligands within the CNS which are recognized by platelets, play an important role in sensing a neuronal damage and participate in the induction and perpetuation of inflammation in the CNS. These ligands are located in the lipid rafts of astrocytes and neurons. We found that platelets bind to the sialated gangliosides integrated into the brain lipid rafts through a number of activating receptors on their surface, such as CD62P, which results in the platelet activation and degranulation.

Our study demonstrates that sialated gangliosides rather than glycoproteins are responsible for the interaction of platelets with brain lipid rafts. First, the treatment of brain lipid rafts with proteases decreased but did not completely inhibit the ability of brain lipid rafts to cause an anaphylactic-like reaction in mice. Second, only the lipid rafts containing sialated gangliosides resulted in an anaphylactic-like reaction. Third, the treatment of lipid rafts with the endoglycosidases that cleave N- and O-glucanes (which deglycosilate proteins) did not inhibit the ability of the lipid rafts to induce anaphylaxis. Fourth, the brain lipid rafts from other mammalian species such as the human and rat also caused an anaphylactic-like reaction in mice.

The composition of sialated glyco-chains in glycoproteins and proteoglycans is highly polymorphic and varies considerably even among closely related species such as human and chimp [50]. Given that there is a substantial difference in the protein structure and glycosylation patterns between mice and human it is unlikely that the human glycoproteins within the CNS lipid rafts would cause anaphylaxis in mice. In contrast, the lipid composition of the mammalian brain is conserved. We found that the brain lipid rafts from an evolutionally remote species such as fish did not cause anaphylaxis in mice. In contrast to mammals, the brain endothelial cells in cartilaginous fishes do not have tight junctions and the blood brain barrier is composed of a tight ring of astrocytes [21], [51]. Therefore, we believe that in the fish both astrocytes and neurons of the neurovascular units do not have specific sialated gangliosides which we found to be recognized by platelets. Indeed, several studies suggested that the lipid composition and structure of gangliosides of the fish brain are different from those of mammals [28]. In addition, we found that both human and mouse lipid rafts caused a serotonin release from both human and mouse platelets *in vitro*. Thus we believe that the recognition of sialated gangliosides by platelets is conserved between the mouse and human, which has direct implications for the development of new therapies for neurodegenerative diseases in patients based on the studies of these structures in murine models.

The mammalian cells are covered with the carbohydrate polymers consisting of glycolipids (such as gangliosides) and glycosylated proteins (glycoproteins and proteoglycans). Together with cholesterol, glycosylated proteins and lipids are organized as stable dense areas of the membranes called lipid rafts. The sialic acid residues are typically the outmost monosaccharide units of both glycolipids and glycoproteins that often serve as the recognition sites to which pathogens (such as influenza virus) attach and infect the target cells [36], [52]. Moreover, many pathogens secrete the neuraminidase that removes sialic acid from the infected cells [53]. Sialation is important for the homeostasis of circulating cells, since it is known that asialated platelets or erythrocytes are eliminated from the blood [54], [55]. Recently, it was shown that asialated proteins and/or cells are recognized by specific cellular receptors (Ashwell-Morell receptors, AMRs) in the liver, and the elimination of asialated platelets in the periphery is an important protective mechanism during systemic infections [53]. It was also reported that the deregulation of the levels of expression of sialic acid and sialated gangliosides on the platelets, cells of blood vessels and tumor cells is associated with other diseases besides the infection, such as a coronary heart disease, cancer metastasis and atherosclerosis [7], [56]–[58]. We found that platelets recognize lipid rafts in the brain, which is known as the most abundant source of sialated gangliosides, such as GM1, GT1b, GD3 and GQ [28]. It has been shown that sialated gangliosides on astrocytes and neurons are important for the formation of an extracellular matrix and myelin structure as well as for the neuronal differentiation [29], [59]. We also found that the lipid rafts in the testis and pancreas (which have a tissue barrier analogous to the BBB) also caused an anaphylactic-like reaction in mice. A large number of glycosphingolipids which were recognized by platelets in our study are found both in the testis and pancreas, including GM1, GD3, GT1b and GQ [60]–[62]. In addition, we found out that the lipid rafts from the heart and lungs induced anaphylaxis. The heart, as well as large blood vessels (such as aorta), contains a large number of gangliosides such as GD3, which cause a platelet adhesion *in vitro* [34]. In addition, lungs are known to contain numerous sialated proteins and glycolipids (GM1, GD3, GD2) on the columnar epithelial cells which serve as receptors for the adhesion of respiratory pathogens [36], [52].

**Figure 7:**
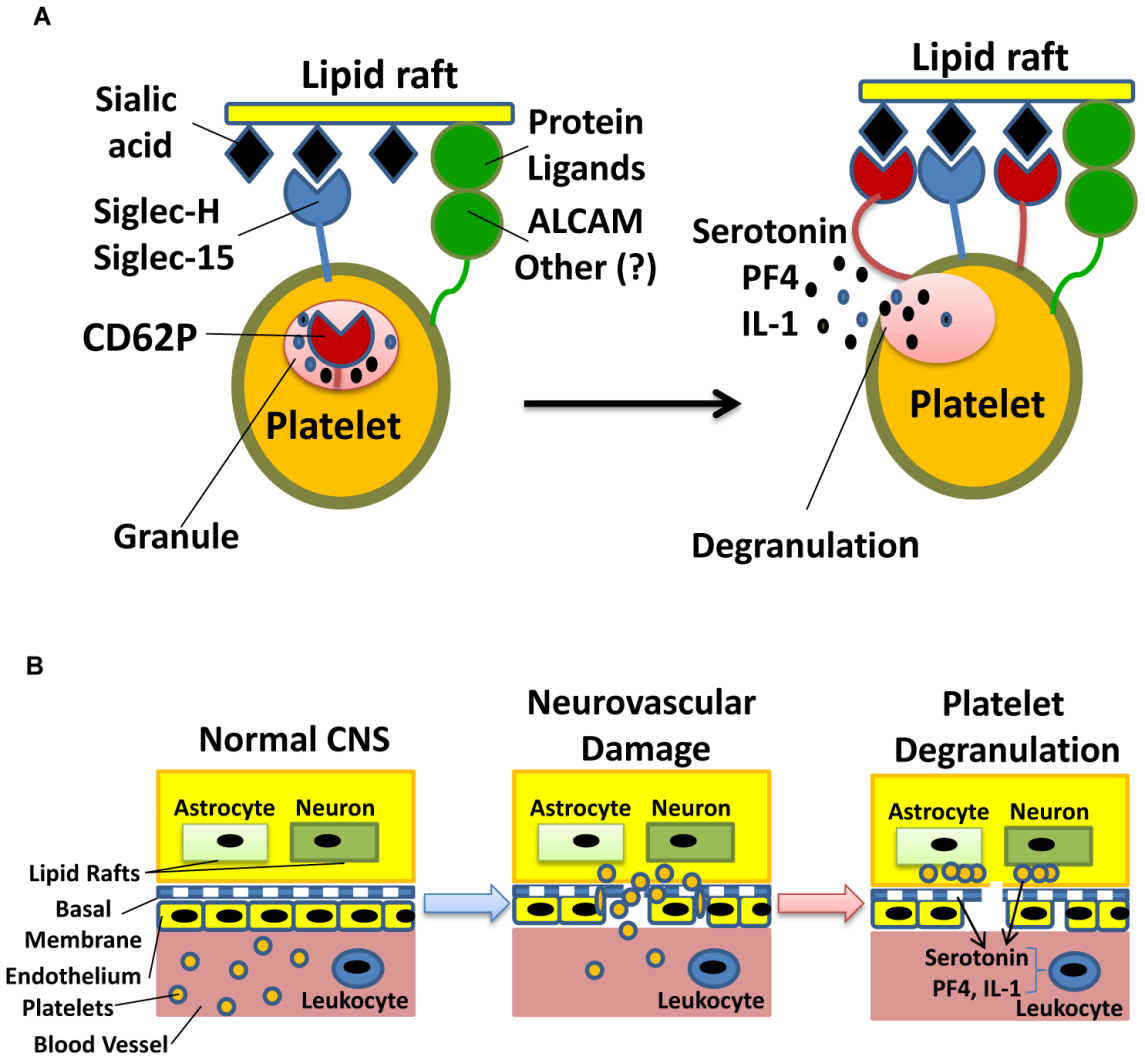
Sialated glycolipids are recognized by platelets as “innate danger signals” of a neuronal damage. (**A**) The proposed model of brain lipid rafts recognition by the platelet receptors. Multiple receptors on the platelets and an intact rigid structure of brain lipid rafts are required to form glycosynapses for the coordinated platelet activation and degranulation.The initial recognition of brain lipid rafts involves the interplay of siglecs and adhesion molecules such as Siglec-H, Siglec-15, ALCAM or other molecules, which results in the recruitment of P-selectin on the surface of platelets to the site of glycosynapse formation and eventual platelets degranulation. (**B**) The schematics of the staged neuronal damage detection process by platelets leading to the recognition of lipid rafts during EAE or neurodegenerative diseases such as MS. In the intact CNS, platelets do not see lipid rafts of astrocytes and neurons due to the impermeable nature of the BBB. The trauma, infection, neurodegeneration or inflammation disrupts the structure of neurovascular units, exposing lipid rafts on the surface of astrocytes and neurons to platelets. The platelets then interact with brain lipid rafts, become activated and secrete proinflammatory cytokines (IL-1) and chemokines (CXCL4/PF4) and serotonin that attract immunocytes to the lesion site and activate them *in situ*. Thus platelets serve as the first line sensors and responders in the early stages of neuronal damage.

Interestingly, CD62P binds ganglioside-specific sialated glycoepitopes (3′-sialyllactose and α2,8-disialic acid), but not others such as lactose or 6′-sialyllactose. At the same time, P-selectin is only one of the receptors that can potentially recognize sialated gangliosides. We propose, that on a molecular level, the gangliosides in the rigid structures of lipid rafts create multiple anchor points with several receptors on the surface of the platelets, known as “glycosynapses” [63] (**Figure 7A**; see also **Supplementary Discussion** in **File S1**). Whereas on a cellular level, brain lipid rafts provide “innate danger signals” for the immune system: the recognition of rafts by platelets results in the platelet degranulation and release of the proinflammatory factors such as serotonin, chemokine CXCL4/PF4, and cytokine IL-1 culminating in the subsequent recruitment of the immune cell subsets (**Figure 7B**). In addition to PF4 and IL-1, serotonin (5-HT) was also found to play a proinflammatory role by activating both innate and adaptive immune cells [64]. Thus we believe that the brain lipid rafts are a new type of the neuronal damage danger signals of great importance, since the multiple broad spectrum agents (NA, CTB, LFA) and specific agents (anti-GQ Abs) that prevented the platelet-raft interactions substantially ameliorated the inflammation in the CNS. The CNS inflammation was also ameliorated in the ST3Gal-V deficient mice that lacked GT1b and GQ aganliosides, further suggesting that the platelet-rafts interactions contribute to the CNS inflammation *in vivo*. The subsequent experiments on the isolation of lipid rafts from the ST3Gal-V^−/−^ and WT mice and the administration into the WT mice or ST3GalV^−/−^ mice will further confirm the role of the gangliosides and platelet-rafts interactions *in vivo*.

Our findings may also have relevance in other disease processes. In Alzheimer’s disease and type II diabetes it was shown that the amyloid precursor protein (APP) peptide directly binds to the gangliosides in the brain lipid rafts and most probably to the lipid rafts in the pancreas [65]–[67]. It is also known that platelets serve as an abundant source of the APP in the human blood [68]. The role of the platelets in the atherosclerosis, heart disease and stroke is well established [69]–[71], and we now identified the sialated gangliosides as new potential platelet ligands in these pathologies. To conclude, our study suggests a novel and important role for the brain lipid rafts and platelets as therapeutic targets in the diseases of the CNS and other pathologies.

## Methods

### Mice

B6 (C57BL/6), B cell deficient µmT (B6.129S2-Igh-6^tm1Cgn^/J), complement deficient C3^−/−^ (B6.C3^−/−^); mast cell deficient Kit^W^/Kit^W–v^ (WBB6F1/J-Kit^W^/Kit^W–v^/J) and +/+ controls from the same colony, CD62P^−/−^ (B6.129S7-Selptm1Bay/J), CD166^−/−^ (B6.129(FVB)-Alcamtm1Jawe/J), ACTB-DsRed transgenic (C57BL/6-Tg(ACTB-bgeo/DsRed.MST), ACTB-GFP transgenic (C57BL/6-Tg(ACTB-EGFP)131Osb/LeySopJ and CD11b-DTR transgenic (B6.FVB-Tg(IGTAM-DTR/EGFP)34Lan/J) mice were purchased from Jackson Laboratories. FcRγ^−/−^ (B6.129P2-Tac-Fcer1<tm1>N12) mice were purchased from Taconic Farms. ST3GalV (*Siat9*) deficient mice were purchased from MMRRC. T/B cell deficient NOD/SCID mice were kindly provided by Dr. Tobias Schatton (BWH, HMS, Boston MA). MOG-TCR transgenic 2D2 mice were maintained in our colony. All animal protocols were approved by Harvard Medical School Animal Care and Use Committee. For experiments that involved pain and/or distress the appropriate anesthetic and analgesic drugs were used. Euthanasia was performed using carbon dioxide.

### Cell Isolation and Cultures

Mouse neuronal (NIE115 and Neuro-2A), astroglial type I (C8-D1A), astroglial type II (C8-S), and brain endothelial (bEnd.3) cell lines were purchased from ATCC. Rat and mouse astroglial and oligodendroglial primary cells were isolated from the mixed glial cultures as described [72], [73]. Mouse oligodendroglial cells were also isolated from the adult mouse brains using OptiPrep Density Gradient [74]. Mouse primary cortical neurons and brain capillary endothelial cells were isolated as described [75], [76].

### Antibodies, Proteins and Reagents

Anti- GQ (A2B5), GT1b, GD, MAP2, GFAP, **galactocerebroside (GC)** mAbs were purchased from Millipore. Fluorophore conjugated anti-CD11b, CD18, CD29, CD41, CD45, CD47, CD54, CD61, CD62P, CD83, CD90, CD106, CD115, were purchased from BD Biosciences. Conjugated anti-mouse CD166 (ALCAM) and MHC class II were purchased from eBiosciences. Non-conjugated anti-ALCAM, anti-CD61 and anti-CD41 antibodies were purchased from R&D Inc, Serotec and BD Biosciences, respectively. Antibodies to MHC class II and CD102 were purchased from Biolegend. Anti-CD36, Siglec-1(CD169), Siglec-4(MAG), PECAM-1 (CD31), Laminin, PDGFR were purchased from Abcam. Anti-Siglec-H and Ly-6G were purchased from eBiosciences. Anti-EAAT-2 (GLT-1) and anti-Siglec-15 antibodies were purchased from Santa Cruz. Since the available anti-Siglec-15 antibodies were not tested by the flow cytometry, we confirmed the expression of siglec-15 in platelets by Western blot prior to flow the cytometry studies.

The rabbit anti-mouse thrombocyte serum was purchased from Accurate Chemical & Scientific Corp. Gangliosides GM1, GM2, GD1b, GT1b were purchased from Sigma. Asialated GM1 (asialoGM1), GD3 and GQ gangliosides were purchased from Calbiochem. Cholesterol, phosphatidylcholine, MβCD (methyl-β-cyclodextrin), CHAPS, Tween 40, Saponin, Triton X-100 and FITC conjugated subunit B of Cholera Toxin (CTB-FITC), Thrombin and Fura-2AM were purchased from Sigma.

The ALCAM-Fc fusion protein was purchased from Leinco Technologies, Inc. The recombinant mouse ALCAM protein (rmALCAM) was purchased from Sino Biological Inc. The CD62P-Fc and PSGL-1-Fc fusion proteins were purchased from R&D.

Lac (Galβ1–4Glcβ-PAA-Biotin), 6′-SLLac (6′-sialyllactose-PAA-Biotin), 3′-SLLac (3′-sialyllactose-PAA-Biotin), α2,8-DiSL (Neu5Aca2-8Neu5Aca-sp-PAA-biotin) biotinylated glycopolymers were purchased from Glycotech Inc.

The enzymes for *in vitro* treatment of lipid rafts (phospholipase C from *B. cereus*, β-galactosidase from *Saccharomyces fragilis*, endo-β-galactosidase from *Bacteroides fragilis*, amyloglucosidase from *Rhizopus sp.,* lipase from human pancreas, sphingomyelinase from *B. cereus,* β*-*glucosidase from almonds, α-Fucosidase from *Xanthomonas sp*., endoglycosidases F1,2,3 from *Elizabethkingia meningoseptica,* sulfatase from *Aerobacter aerogenes*) were purchased from Sigma. Neuroaminidase (NA) and non-conjugated Cholera Toxin B subunit (CTB) used for *in vivo* administration in mice with EAE were purchased from Sigma. *Limax flavus* agglutinin (LFA) was purchased from EY Laboratories Inc. To treat mice with EAE, NA (0.4 U/mouse), CTB (40 µg/mouse), LFA (0.4 mg/mouse) were injected i.p. on day 0, 2, 4, 6 and 8 after EAE induction. Anti-thrombocyte serum was injected i.v. on day 0 and 2 (30 µl/mouse) and then i.p. (40 µl/mouse) on day 4 and 8 after EAE induction. Anti-GQ (A2B5) Abs were injected i.v. (40 µg/mouse) on day 0, 2, 4, 6, 8, 10, 12 and 14 after EAE induction.

For the depletion of cells *in vivo* 0.5 mg/mouse of antibodies (injected i.p) or 30 µl of the anti-thrombocyte serum (injected i.v.) were used. Antibodies were injected i.p. 24 hours or i.v. 4 hours prior to the analysis. The extent of depletion of platelets by the anti-thrombocyte serum or anti-CD61 mAbs was assessed by counting the platelets in the peripheral blood. Platelets counts were decreased with both antibodies from 5–8 to 0.1–0.3 (×10^5^/µl). The granulocyte depletion was performed by the treatment with anti-Ly-6G antibodies. The granulocyte counts in the peripheral blood were assessed by microscopy after the lysis of erythrocytes, fixation of the cells with methanol and H&E staining. The granulocyte numbers were decreased from 5–7 to 0.03–0.1 (×10^6^/ml). For blocking of the receptors *in vivo* 0.25 mg/mouse of antibodies were injected i.v. 30 minutes prior to the injection of lipid rafts. For the depletion of macrophages in CD11b-DTR transgenic mice [77], 500 ng/mouse of diphtheria toxin (DTx) was injected i.p. every other day for 12 days. The extent of macrophage depletion by DTx was assessed by FACS by measuring the percentage of CD11b^+^F4/80^+^ cells in the peritoneal cavity and in the peripheral blood. We found that the percentages of CD11b^+^F4/80^+^ cells decreased from 46±5% to 6±2% in the peritoneal cavity and from 8±3% to 2±1% in the peripheral blood. The depletion of macrophages with liposomes with bisphosphonate clodronate was performed as described [78]. We found that the percentage of CD11b^+^F4/80^+^ macrophages decreased from 7±2% to 1±1% in the peripheral blood as determined by FACS.

### Isolation of Lipid Rafts

Lipid rafts from the mouse brain, spinal cord, liver, lungs, testis, eyes, thymus, spleen, kidney, ovaries, pancreas, adrenal glands and heart were isolated by the homogenization of 0.2 mg of wet tissue per 1 ml of PBS with 0.5% Triton X-100 (Sigma). After which, the homogenate was centrifugated at low speed (250 g) for 5 minutes and the supernatant was filtered through 0.4 µ and nylon filter (Millipore) and washed two times with PBS by the centrifugation at high speed (16,000 g) for 10 minutes. The rafts were injected i.v. 100 µl per mouse. The lipid rafts were also obtained by a detergent-free method using recently published protocol with modifications [79]. Briefly, 0.5 mg of the brain wet tissue was homogenized in 1 ml of PBS, and after the centrifugation at 400 g for 10 min, the supernatant was filtered through 0.4 µ and then through 0.2 µ filter (Millipore) and injected i.v. 200 µl per mouse. In some experiments, lipid rafts were isolated from the cultured cells, frozen samples of the human brain, rat brain, or frozen fish brain (Atlantic Cod and Shark), as described above. To purify the lipid rafts from the human white and gray matter of the brain, unidentified frozen autopsy samples from New York tissue bank were used. The work with human autopsy samples was approved by HMS review board committee. The gray and white matters were dissected, the lipid rafts were isolated by the homogenization in PBS with 0.5% Triton X-100. To isolate the lipid rafts from a myelin fraction of the rat brain (Lewis rats), the myelin fraction of the homogenized rat brain was isolated using a sucrose gradient and ultracentrifugation as described [80], and after that the lipid rafts were prepared using PBS with 0.5% Triton X-100. The amount of lipid rafts was quantified using a kit for the measurement of the concentration of phospholipids (Wako Chemicals) and injected i.v. at 1 µg of phospholipids per mouse.

### Fractionation and Quantification of Lipid Rafts

Lipid rafts were isolated using PBS with 0.5% of Triton X-100 and analyzed using a 40%/35%/5% discontinuous sucrose gradient and ultracentrifugation (38,000 g for 19 hours) on the Beckman ultracentrifuge [79], [81]. Four or 11 fractions were collected and the concentration of phospholipids, cholesterol were assessed using the kits from BioVision and Wako Chemicals and the amount of the transferin receptor (Tfr) was assessed by Western blot analysis.

### Assessment of the Anaphylactic-like Reaction in Mice

The mice were restrained and injected i.v. with the indicated amounts of lipid rafts and immediately released. The mice were observed for 10 minutes when the clinical scores and the body temperature were assessed and then overlooked for an additional hour to ensure their recovery. The clinical scores were assessed as follows: 1) restless behavior; 2) loss of consciousness; 3) dyspnea; 4) death. The body temperature was measured using a digital precision thermometer equipped with a rectal probe (YSI Sensors&Instruments). The positive anaphilactogenic effect was registered at a score of one or higher. When we monitored the symptoms during a ten-minute period, we observed that the clinical symptoms typically reached the maximum in five minutes after the administration (**Figure S1a** in **File S1**) after which the mice started to recover. The anaphylactic symptoms were also accompanied by hypothermia (**Figure S1b** in **File S1**), a known feature of an anaphylactic shock. After a 10-minute observation the mice were administrated with analgesic agents and saline, and monitored for several hours to ensure complete recovery. The histamin level was measured in the plasma in a group of 5–7 unmanipulated mice and in a group of 5–7 mice after the administration of lipid rafts using an ELISA kit from GenWay.

### Model Lipid Rafts

The model lipid rafts were prepared as described [82] with modifications. Briefly, 1 mg of a particular ganglioside (asialoGM1, GM1, GD1b, GD3, GM2, GT1b, GQ), or 1 mg of galactosylcerebroside (GC) or 1 mg phosphatidylcholine (used as a background control, Bkg) were mixed with 0.5 mg of phosphatidylcholine and 6 mg of cholesterol, dissolved in 1 ml of chloroform/methanol (1∶1) and allowed to dry on a SpeedVac for 1–2 hrs. The lipids were then resuspended in 1 ml of PBS with 0.5% Triton X-100, vortexed (5 min) and sonicated (3 min) several times until a homogeneous suspension is obtained. The suspensions were centrifuged at 16,000 g for 10 min, the supernatants were completely decanted and pellets were resuspended in 1 ml of PBS. The suspension of model rafts was injected i.v. 250 µl/mouse or used for the *in vitro* serotonin release assay described below.

### Platelet Isolation and Analysis

For the isolation of platelets from the periphery, the blood was collected and 0.38% sodium citrate was added to the tubes to prevent coagulation. The platelet rich plasma (PRP) was obtained by a low speed centrifugation (250 g) for 5 minutes. The PRP was used for the Flow Cytometry and serotonin release assay. For the FACS analysis, the percentage of CD41^+^CD61^+^ platelets was analyzed in the forward/side scatter “platelet” gate as described [8]. For the platelet adhesion assay and Western blot analysis, the platelets were further purified by filtration of the PRP through a Sepharose 40 (Sigma) column with BSA as described [83].

For the isolation of platelets from the CNS, the mice were perfused with PBS and the brains or spinal cords were dissected, homogenized, and centrifuged at a low speed (250 g) for 5 minutes. The Platelet rich supernatant of the CNS homogenate was used for the FACS analysis of platelets similarly to the FACS analysis of platelets in the PRP by determining the percentage of CD41^+^CD61^+^ platelets in the “platelet” forward/side scatter gate.

### Platelet Serotonin, PF4/CXCL4 and IL-1α and Release Assays

300 µl of the platelet rich plasma were mixed with 60 µl of the brain lipid rafts or PBS and incubated for 30 minutes for serotonin and 1–4 hours for PF4 and IL-1α at +37°C. Then the samples were centrifuged at 16,000 g for 7 minutes at +4°C and 100 µl supernatant (platelet-free plasma) was used for serotonin (GenWay), mouse PF4/CXCL4 (Uscn Life Science) or IL-1α Biolegend) ELISAs according to the manufacturer’s recommendations. For the blocking experiments, 250 µl of the platelet rich plasma were mixed with 50 µl of the blocking antibodies or PBS and incubated at +20°C for 15 min before the addition of the brain lipid rafts.

### Platelet Adhesion Assay

The adsorption of gangliosides to a 96 well flat bottom plate was performed as described [84]. Briefly, gangliosides were mixed with cholesterol, dissolved in ethanol, added 150 µl/well to the plate and allowed to dry overnight at 20°C. The next day the plates were first washed with water and then in Tyrode solution (137 mM NaCl; 2.8 mM KCl; 1 mM MgCl_2_; CaCl_2_ 2 mM; 0.4 mM NaH_2_PO_4_; 12 mM NaHCO_3_; 5.5 mM glucose; 5 mM HEPES 0.35% BSA, pH = 7.4). After that the plates were incubated with Tyrode solution 100 µl/well for 2 hrs at 37°C before the addition of platelets. The platelets were isolated by filtration of the PRP from the DsRed tg mice through a Sephadex 40 column saturated with PBS and added to the plates with adsorbed ganagliosides as described [34]. After 2 hours of incubation at 37°C the plate was gently washed three times with a Tyrode solution and the fluorescence was measured on the TECAN multichannel spectrofluorometer.

### EAE Induction

EAE was induced by a subcutaneous immunization with 150 µg MOG in 4 mg/ml CFA of 8–12–week-old B6 mice as was described previously [85]. Pertussis toxin (PTx) was given i.p. (150 ng/mouse) on days zero and two post-immunization. The mice were observed for the signs of disease starting on day five post-transfer, and the disease severity was scored on a numerical scale 0–5 as follows: 0) no disease; 1) weak tail or wobbly walk; 2) hind limb paresis; 3) hind limb paralysis; 4) hind and forelimb paralysis; and 5) death or euthanasia due to humane reasons.

When we examined the mice with EAE after the platelet depletions, we did not find signs of spontaneous hemorrhages in the brain, spinal cord or other organs except for the intestines and skin, where bleeding was observed in 30–40% of mice. A similar percentage of mice with depleted platelets that were immunized with CFA alone also had intestinal/skin bleeding which was associated with ∼30% mortality. To decrease the rate of mortality we lowered the dose of CFA from 4 mg/ml to 3 mg/ml and kept the same amount of MOG peptide and PTx. The decrease in CFA eliminated the symptoms of skin and intestinal bleeding and decreased the rate of mortality to 5–10%, a level similar to the non-immunized mice with depleted platelets. The decrease of CFA for immunization slightly decreased the clinical EAE scores in the control group of mice from a typical score of 2 to a score 1.5 at the peak of EAE, but did not affect the incidence or the onset of the disease.

For the induction of EAE without PTx, the mice immunized with MOG were injected 75 µl/mouse i.v. of brain lipid rafts or PBS; or were injected i.c. 20 µl/mouse PRP or PBS on day 0 post-immunization.

### Flow Cytometry

The multicolor flow cytometry analysis was conducted in the Flow Cytometry core facility of The Center for Neurologic Diseases (Brigham and Women’s Hospital, Harvard Medical School) following the standard procedures. The isolation of cells from the CNS and spleen and the flow cytometry analysis were performed as described [85]. The flow cytometry analysis was conducted on the LSR II Cytometer. The imaging cytometry was performed on the ImageStream™ cytometer (Amnis Inc.) in Flow and Imaging Core Facility of Immune Disease Institute (Harvard Medical School). The images of cells were analyzed using the Imagestream Data Exploration and Analysis Software (IDEAS).

For the analysis of brain lipid rafts, the rafts were centrifuged at 16,000 g for 10 minutes and resuspended in the 70% goat serum in PBS for blocking of the non-specific staining. After 30 min of incubation in the goat serum, FITC conjugated CTB (that stain GM1), anti-GM2, anti-GD1b, anti-GD3 anti-GT1b, anti-A2B5 (that stain GQ), anti-GC, or anti-GLT-1 antibodies were added to the samples and incubated for 20 min at room temperature, washed once with PBS, after which the lipid rafts were centrifuged at 16,000 g for 10 minutes, resuspended in PBS with the 70% goat serum and secondary antibodies and incubated for 20 minutes at room temperature. Finally the samples were washed twice with PBS and fixed in 1% paraformaldehyde in PBS and analyzed on the LSR II cytometer. For the FACS analysis of lipid rafts, we adapted a published method for the detection of small viral particles of the sizes less than 200 nm [86]. The FSC and SSC parameters were set to a logarithmic scale and the threshold decreased. To ensure we did not detect the noise in our FSC/SSC gate, we used staining for GM1 with CTB-FITC, which gave a very bright fluorescent signal in the FL-1 channel. We found that 97–99% of the lipid rafts in the FSC/SSC gate were GM1 positive.

For the analysis of platelets from the periphery, 1 µl of anti-CD41, anti-CD61 and either of other surface marker antibodies (CD54, CD62P, CD83, CD102, CD106, CD166, CD169, Siglec-H, Siglec-4) at proper dilution were added to 200 µl of PRP, incubated 20 min at +20°C, the cells were fixed by adding of 1 ml of 1% paraformaldehyde in PBS and analyzed by the three-color flow cytometry. For the analysis of platelets from the brain or spinal cord, the brains or spinal cords were homogenized and centrifuged at a low speed (250 g). After that, 200 µl of the platelet rich supernatant of the CNS homogenate was mixed with 200 µl of the normal goat serum and incubated at +20°C for 15 min, after which 3 µl of anti-CD41 and anti-CD61mAbs were added. After the incubation with antibodies for 10–15 min at +20°C, the cells were fixed in 1 ml of 1% paraformaldehyde in PBS. For the assessment of platelets and platelet-derived microparticles, the forward and size scatter parameters on the flow cytometer were set to a logarithmic scale and the CD61^+^CD41^+^ gated cells in the “platelet” forward/size scatter gate were analyzed as platelets, and the CD41^+^ particles in the sub-platelet “microparticle” forward/size scatter gate were determined as platelet-derived microparticles as described [8].

### CD4 T cell Recall Response

The MOG-TCR transgenic 2D2 mice were immunized with 150 µg MOG in 4 mg/ml CFA, and seven days later the CD4^+^ T cells were isolated from the spleen by negative selection using magnetic beads as described [85]. Anti-GQ (A2B5) mAbs or PBS were injected i.v. (40 µg per mouse) on day 0, 2, 4 and 6 post-immunization. The CD4 T cells were incubated with irradiated splenocytes from the B6 mice for 48 hours in the presence of MOG_35–55_ (5–20 µg/ml) with or without anti-GQ mAbs (5–10 µg/ml), after which the level of proliferation was assessed by the flow cytometry examining the bromodeoxyuridine (BrdU) labeling of the cells *in vitro* as described previously [85].

### Platelet Transmigration Assay

The brain endothelial cell line (bEnd.3) was grown for 72 hours to reach the confluence in the top chamber of the transwell system (the membrane inserts) for 24 well plate with 5 µm pore size (Corning). The astroglial cells (C8-D1A) were grown in the bottom chamber at the density close to the confluent for 72 hours. The barrier integrity was checked by a fluorescently labeled dextran flux to the lower chamber and measured using a spectrofluorometer as described [41]. The platelets isolated from the ACTB-GFP transgenic mice by centrifugation were added to the top chamber. For the activation of endothelial cells PTx (200 ng/ml) and/or MOG (40 µg/ml) with CFA (50 µg/ml) were added to the cultures as described [41] simultaneously with the platelets. After the incubation in the CO_2_ incubator for 18 hours, photographs from the bottom chambers were taken using an inverted fluorescent microscope. After that the GFP^+^ platelets were washed from the astroglial monolayer, the supernatant was plated at 200 µl/well in triplicate in a flat bottom 96 well plate for the fluorescence measurement and read on a TECAN spectrofluorometer (excitation at 488 nm, emission at 525 nm).

### CD62P (P-selectin) and CD166 (ALCAM) Binding Assays

To assess the ability of CD62P and CD166 to bind sialated glycoepitopes, streptavidin was adsorbed to flat bottom 96 well high-binding plates (Nunk), and after washing and blocking with TBS buffer with 2% BSA, Lac, 6′-SLLac, 3′-SLLac and α2,8-DiSL biotinylated glycopolymers were added at 10 µg/ml to the plate. After several washes, the CD62P-Fc or CD166-Fc fusion proteins (at concentrations of 0–5 µg/ml) were incubated with the immobilized probes. After the incubation and washing, the antibodies that recognize a human Fc fragment conjugated with AP (Acris) were used for detection. Finally the plates were incubated with an AP substrate and analyzed on a TECAN spectrophotometer (absorbance at 405 nm). To assess the of ability of CD62P to bind ALCAM, the recombinant mouse ALCAM protein was adsorbed to the plate, and CD62P-Fc and the antibodies that recognize the human conjugated with AP Fc fragment were used as described above.

### Immunofluorescent Microscopy

The normal mice or mice with EAE were perfused first with PBS, and then 1% of paraformaldehyde in PBS. The brains/spinal cords were dissected from the mice or human brain tissue samples and fixed in 1% of paraformaldehyde in PBS overnight, and then dehydrated in 30% sucrose for 2–3 days. 10 µ-thick frozen sections were prepared and stained for GM1 (with CTB-FITC or CTB-biotin combined with SA-AF594), and GFAP, or MAP2, or Laminin, or CD31, or PDGFR or CD41 combined with the secondary antibodies conjugated with AF594.

### Statistical Analysis

Student’s t-test was used to validate the significance of the observed differences. A p-value of less than 0.05 was considered statistically significant.

## Supporting Information

File S1 **Combined Supplementary Information File.** Supplementary Tables (Tables S1–S5), Supplementary Figures (Figures S1–S16), and Supplementary Discussion with Supplementary References.
(PDF)

Video S1 **Clinical symptoms of the anaphylactic-like reaction induced by the brain lipid rafts.** Video starts 45 seconds after i.v. administration of brain lipid rafts.
(WMV)

Video S2 **Clinical symptoms of typical thromboembolism.** Video starts 45 seconds after i.v. administration of thrombin.
(WMV)
