# Correction: Platelets Recognize Brain-Specific Glycolipid Structures, Respond to Neurovascular Damage and Promote Neuroinflammation

**DOI:** 10.1371/annotation/0bbea8d3-1f94-48af-915c-aec02da2f5c3

**Published:** 2014-01-08

**Authors:** Ilya Sotnikov, Tatyana Veremeyko, Sarah C. Starossom, Natalia Barteneva, Howard L. Weiner, Eugene D. Ponomarev

In Figure 6, the labels in Figure 6C and Figure 6F are mixed up. Please see the corrected Figure 6 here: 

**Figure:**
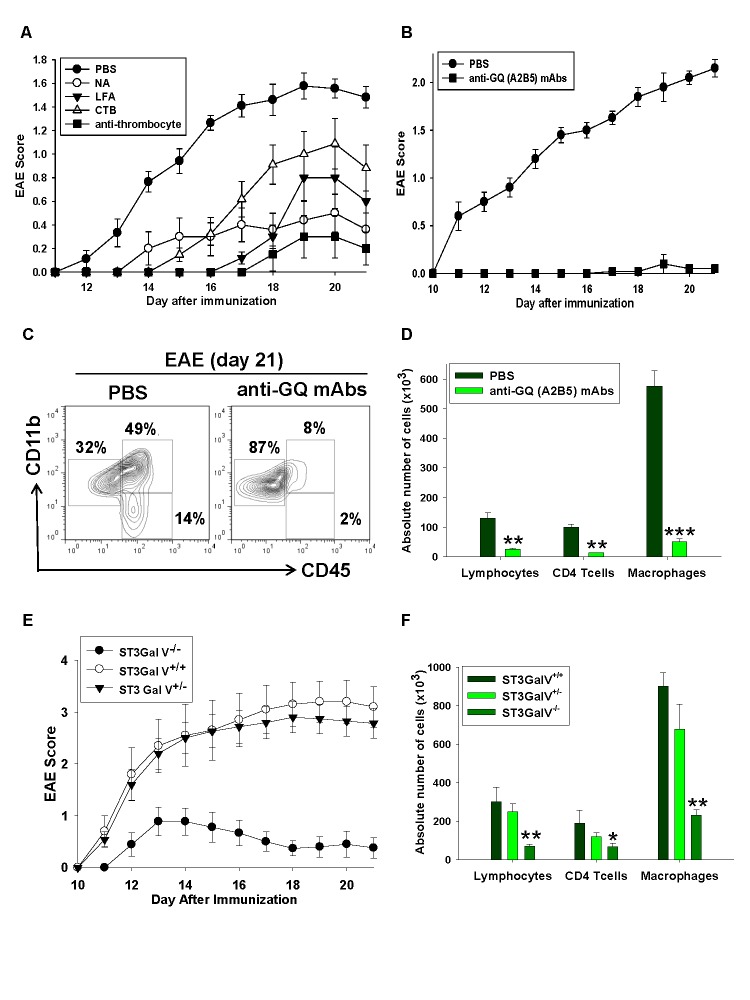


f

In the Supporting Information file S1: Table S3, the columns in the "Time to death" row are swapped. Please see the corrected File S1 here: 
