# Supplementary material for: Correction: Platelets Recognize Brain-Specific Glycolipid Structures, Respond to Neurovascular Damage and Promote Neuroinflammation
Source: PLoS One. 2014 Jan 8;9(1):10.1371/annotation/0bbea8d3-1f94-48af-915c-aec02da2f5c3. doi: 10.1371/annotation/0bbea8d3-1f94-48af-915c-aec02da2f5c3 (PMC5703086; doi:10.1371/annotation/0bbea8d3-1f94-48af-915c-aec02da2f5c3)
Supplement: Supplementary file 1 [file 0bbea8d3-1f94-48af-915c-aec02da2f5c3.s001.pdf]

<u>SUPPLEMENTARY INFORMATION</u>

(Supplementary Tables, Figures, Discussion and References)

Platelets recognize brain-specific glycolipid structures, respond to neurovascular damage and promote neuroinflammation

Ilya Sotnikov, Tatyana Veremeyko, Sarah-Christin Starossom, Natalia Barteneva, Howard L. Weiner and Eugene D. Ponomarev

<u>Table S1.</u> Depletion of platelets and white blood cell populations and its effect on the ability of brain lipid rafts to cause an anaphylactic-like reaction[1]

Depleted Population	Method of depletion	Anaphylactic-like reaction	N
**Mast cells**	Mast cell-deficient mice ($Kit^{W/W-v}$)	+	10
**B cells**	B cell-deficient mice (μmT)	+	10
**T and B cells**	T/B cell deficient mice (NOD/SCID)	+	5
**Macrophages[2]**	CD11b-DTR tg mice, DTx[3] (i.p.)<br><br>Lyposomes with bisphosphonate clodronate (i.v.)	+<br><br>+	5<br><br>5
**Granulocytes[2]**	anti-Ly6G mAbs (NA/LE[4])	+	5
**Platelets[2]**	anti-CD61 (NA/LE[4]) mAb<br><br>anti-thrombocyte serum	-<br>-	5<br>10

[1]Mice were injected i.v. with 100 μl/mouse brain lipid rafts isolated by the homogenization of brain in PBS with 0.5% Triton X-100 and the symptoms of anaphylaxis were observed as described in *Methods*.

[2] The nidicated populations of cells were depleted as described in *Methods.*

[3]DTx - diphtheria toxin.

[4]NA/LE – no sodium azide/low endotoxin.

Table S2. **Analysis of the composition and ability of brain lipid rafts to cause an anaphylactic-like reaction[1]**

Treatment	Treatment Conditions (temperature and time)	0.2 µ filtered supernatant (0.5 mg/ml, 200 µl/mouse)			Triton X-100 insoluble particles (0.2 mg/ml, 100 µl/mouse)		
		Effect[2]	Maximal Score[3]	N	Effect[2]	Maximal Score	N
**No Treatment**	-	+	**2.3 ±0.2**	12	+	**2.3± 0.1**	15
**Boiling**	100 °C, 10 min	-	**0***[4]**	3	+	**0**	5
**Sonication**	20 °C, 45 min	+	**1.7 ± 0.3**	3	**ND[5]**	**ND**	ND
**Tween 40 (0.5%)**	20 °C, 30 min	-	**0*** **	6	-	**1 ± 0.4** **	4
**CHAPS (0.5%)**	20 °C, 30 min	-	**0*** **	6	-	**0***** **	3
**Trypsin (0.25%)**	37 °C, 1 hr	+	**1.7 ± 0.3**	3	+/-	**1.3 ± 0.3* **	3
**Saponin (0.5%)**	20 °C, 1 hr	**-/+**	**0.4 ± 0.2***** **	12	+/-	**0.6 ± 0.2***** **	9
**Proteinase K (30-50 U/ml)**	37 °C, 2.5 hrs	+	**2.3 ± 0.5**	12	-	**0.9 ± 0.1****** **	14
**Sphingomyelinase (30-50 U/ml)**	37 °C, 2.5 hrs	-	**0***** **	6	-	**0****** **	6
**Phospholipase C (30-50 U/ml)**	37 °C, 2.5 hrs	-	**0*** **	3	-	**0***** **	3
**Lipase (30-50 U/ml)**	37 °C, 2.5 hrs	**ND**	**ND**	ND	-	**0***** **	3
**Collagenase (0.1%)**	37 °C, 2.5hrs	**ND**	**ND**	ND	+	**1.6 ± 0.2**	7
**MβCD (20 nM)**	37 °C, 1hr	**-/+**	**0.4 ± 0.2****** **	14	+/-	**0.7 ± 0.2****** **	9
**α-Galactosidase (10 U/ml)**	37 °C, 8hr	**ND**	**ND**	ND	-	**1.8 ± 0.3**	3
**β-Galactosidase (20 U/ml)**	37 °C, 4 hrs	**+/-**	**1 ± 0.3** **	5	-	**0.2 ± 0.1********* **	15
**Endo-β-Galactosidase (20 U/ml)**	37 °C, 4 hrs	**ND**	**ND**	ND	-	**0**** **	3
**β-Glucosidase (20 U/ml)**	37 °C, 4 hrs	**ND**	**ND**	ND	+	**2.6 ± 0.7**	3
**Amyloglucosidase (10 U/ml)**	37 °C, 8 hrs	**ND**	**ND**	ND	+	**2.3 ± 0.6**	4
**Sulfatase (20 U/ml)**	37 °C, 4 hrs	**ND**	**ND**	ND	+	**2.6 ± 0.2**	8
**α-Fucosidase (2 U/ml)**	37 °C, 24hrs	+	**2.0 ± 0**	3	+	**2.6 ± 0.4**	5
**Endoglycosidase F1 (2 U/ml)**	37 °C, 24 hrs	**ND**	**ND**	ND	+	**1.8 ± 0.2**	3
**Endoglycosidase F2/F3 (1 U/ml each)**	37 °C, 24 hrs	**ND**	**ND**	ND	+	**2.5 ± 0.8**	3
**Neuraminidase (2 U/ml)**	37 °C, 16 hrs	-	**0*** **	3	-	**0**** **	5

[1]The brain lipid rafts were isolated as described in *Methods* and injected i.v. at indicated doses.

[2]The anaphylactic-like reaction was assessed as described in *Methods.*

[3]The maximal anaphylactic score (mean ± S.E) observed within 10 min after the administration of brain lipid rafts is shown.

[4]**, p<0.01; ***, p<0.001; ****, p<0.0001; *****, p<0.00001; ******, p<0.00001; *********, p<0.000000001 when compared to non-treated control.

[5]ND – not determined.

Table S3. **Comparison of symptoms and manifestations of the anaphylactic-like reaction induced by the intravenous administration of brain lipid rafts and the thromboembolism induced by the intravenous administration of thrombin[1]**

Symptoms/manifestation	Anaphylactic-like reaction induced by brain lipid rafts	Thromboembolism induced by thrombin
Ameliorated by anti-histamine dugs[2,3]	yes	no
Necrotic areas in lungs in postmortem examined animals[2,3]	no	yes
First symptoms of abnormal behavior[2]	in 1-3 min after administration	immediately
Loss of consciousness and spontaneous recovery in 10-15 min[3]	yes	no
Time to death[2]	8-10 min	1-3 min
Rescue form death by i.v. injection of 1 ml of saline[2]	yes	no
Tissue swelling	yes	no
Body temperature drop[3,4]	+++	+
Platelet count drop[3,5]	+++	+
Bleeding time[3,6]	Increases[7]	Decreases[8]
Formation of platelet-derived microparticles[3.9]	++	+

[1]100-150 μl of brain lipid rafts or 0.6-1.25 kU/kg of thrombin dissolved in 100-150 μl of saline were injected i.v. and the mice were observed for clinical symptoms and other parameters.

[2]Lethal dose (brain lipid rafts: 150μl/mouse; thrombin: 1.25 kU/kg).

[3]Sublethal dose (brain lipid rafts: 100μl/mouse; thrombin: 0.6 kU/kg).

[4]The temperature drop within 4 minutes for brain lipid rafts is $\Delta T=3.8\pm0.1\ ^{o}C$ while for thrombin it is $\Delta T=0.8\pm0.1^{o}C$.

[5]The average platelet count drop was 4.8-fold for the lipid rafts treatment and 1.4-fold for the thrombin treatment

[6]Atail bleeding test was performed 4 minutes after the administration of the tested substances in a form of tail transection(2 mm from the mouse tail tip) with a subsequent immersion into PBS.

[7]The tail bleeding time for the mice injected with lipid rafts was 390 ± 46 s, while for the mice injected with saline it was 126 ± 14 s.

[8]The tail bleeding time for the mice injected with thrombin was 37 ± 4 s, while for the mice injected with saline it was 126 ± 14 s.

[9]The percentage of $CD41^{+}$ microparticles increased 2.9-fold for lipid rafts and 1.2-fold for thrombin

Table S4. **Comparison of the potency of lipid rafts from different organs to cause an anaphylactic-like reaction[1].**

Organ	Relative amount of lipid rafts compared to brain	Normalized for amount of wet tissue (20 μg/mouse)[2]			Normalized for the amount of phospholipids (1 μg/mouse)[3]		
		Effect	Maximal Score[4]	N	Effect	Maximal Score	N
Brain	1	+	**2.2 ± 0.2**	9	+	**2.3 ± 0.1**	9
Spinal Cord	0.7	+	**1.7 ± 0.2**	6	+	**2.0 ± 0.3**	9
Liver	0.3	-	0	6	-	0	9
Lungs	0.15	-	0	9	+/-	**0.6 ± 0.2**	14
Testis	0.4	-	0	9	+	**1.3 ± 0.4**	12
Eyes	0.07	-	0	5	-	0	9
Thymus	0.05	-	0	6	-	0	9
Spleen	0.2	-	0	6	-	0	9
Kidney	0.3	-	0	6	-	0	9
Ovaries	0.03	-	0	5	-	0	9
Pancreas	0.2	-	0	5	+/-	**0.7 ± 0.3**	15
Adrenal	0.1	-	0	3	-	0	4
Heart	0.1	-	0	3	+	**2.0 ± 0.4**	3

[1]Lipid rafts were isolated by the homogenization of organ tissues in PBS with 0.5% Triton X-100 as described in *Methods.*

[2]100 μl of lipid rafts were injected i.v. per mouse.

[3]The amount of phospholipids was determined and the concentrations of lipid rafts from various organs were adjusted to the concentration of phospholipids of the lipid rafts from brain (10 μg/ml); 100 μl/mouse of the lipid rafts (1 μg of phospholipids per mouse) were injected i.v.

Table S5. **The role of the known receptors and factors important for the recognition of sialated gangliosides in natural (brain) and artificial (model) lipid rafts by platelets[1].**

Receptor/ molecule	Method of blocking[2]	Brain lipid rafts		Model lipid rafts	
		Effect[3]	N	Effect	N
**β1 integrin (CD29/GPIIa)**	Ha2/5 mAbs (BD Biosciences 555002)	+	4	+	3
**β2 integrin (CD18)**	M18/2 (553341) and GAME-46 (BD Biosciences 555280) mAbs	+	4	+	3
**β3 integrin (CD61/GPIIIa)**	$\beta3^{-/-}$ mice	+	10	+	3
**FcRγ**	$FcR\gamma^{-/-}$ mice	+	10	+	3
**CD36/GPIV**	MF3 mAbs (Abcam ab80080)	+	4	+	3
**Compliment**	$C3^{-/-}$ mice	+	10	+	3

[1]Lipid rafts were isolated from the brain tissue using Triton X-100, and the model lipid rafts containing ganglioside GT1b were prepared as described in *Methods.*

[2]For blocking of specific molecules genetically deficient (knockout, -/-) mice were used, or antibodies were injected i.v. prior to the i.v. injection of lipid rafts as described in *Methods.*

[3]The anaphylactic reaction was assessed as described in *Methods.*

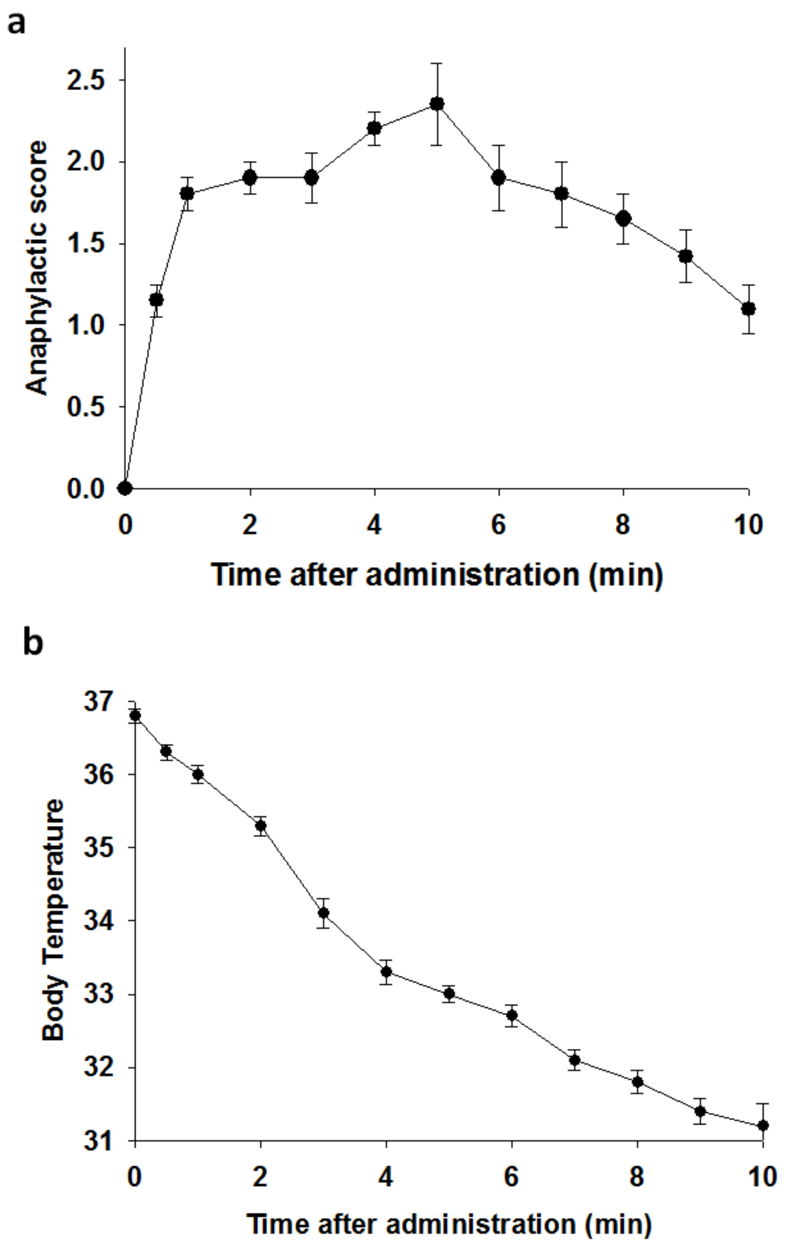

Figure S1. **<u>Clinical manifestations of the anaphylactic-like reaction in mice after i.v. administration of brain lipid rafts.</u>**

Mice were injected i.v. with the brain lipid rafts obtained by the homogenization of brain tissue in PBS with 0.5% Triton X-100 as described in *Methods.* The anaphylactic-like reaction (see *Methods* for details) was observed during a ten-minute period and scored as follows: 1) restless behavior; 2) loss of consciousness; 3) dyspnea; 4) death. The anaphylactic score (**a**) and body temperature (**b**) with mean ± S.E. of the total number of animals from the five experiments with groups of five mice per experiment is shown.

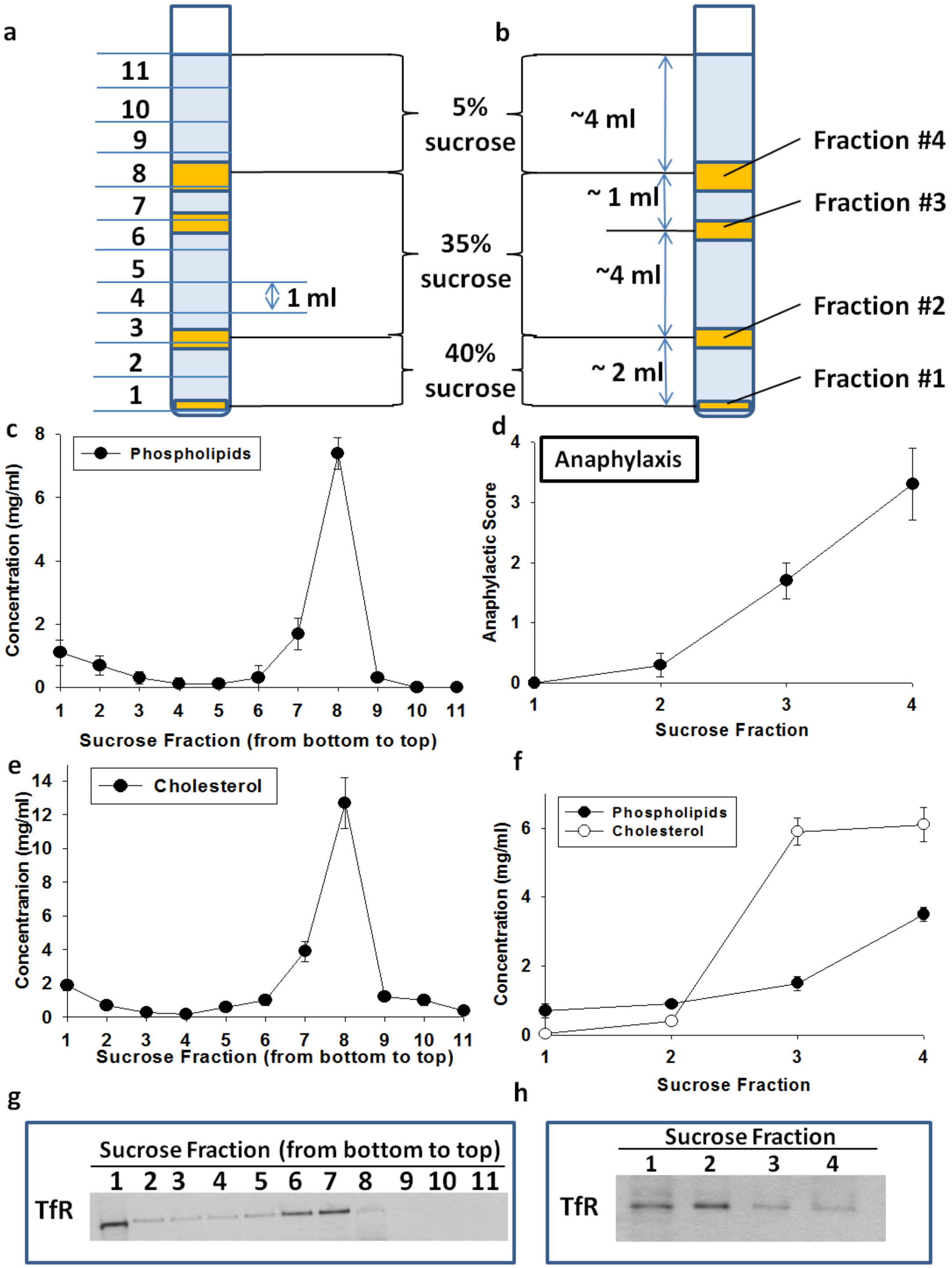

Figure S2. <u>Fractionation of brain lipid rafts.</u> Lipid rafts were isolated by the homogenization of brain tissue in PBS with 0.5% Triton X-100, overlayed on a 40%/35%/5% sucrose discontinuous gradient and centrifuged at a high speed as described in *Methods*. The total of the 11 fractions shown in (**a**) were analyzed by taking 1 ml portions from the centrifuge tube (fractions are numbered from bottom to top) and by measuring the concentration of phospholipids (**b**) and cholesterol (**d**) in each fraction. Four bands were also identified visually in the centrifuge tube as shown in (**b**), separated, washed with PBS and re-suspended in equal volumes identical to the initial volume of non-separated brain lipid rafts. For each of the four fractions identified in (**b**), anaphylaxis was tested by the i.v. injection of 100 µl/mouse of fractionated lipid rafts (**c**) and the concentrations of phospholipids and cholesterol were measured (**e**). The amount of the transferin receptor (TfR) was measured by Western blot for all the 11 fractions (**g**) and for the four fractions (**b**) identified visually **(h)**.

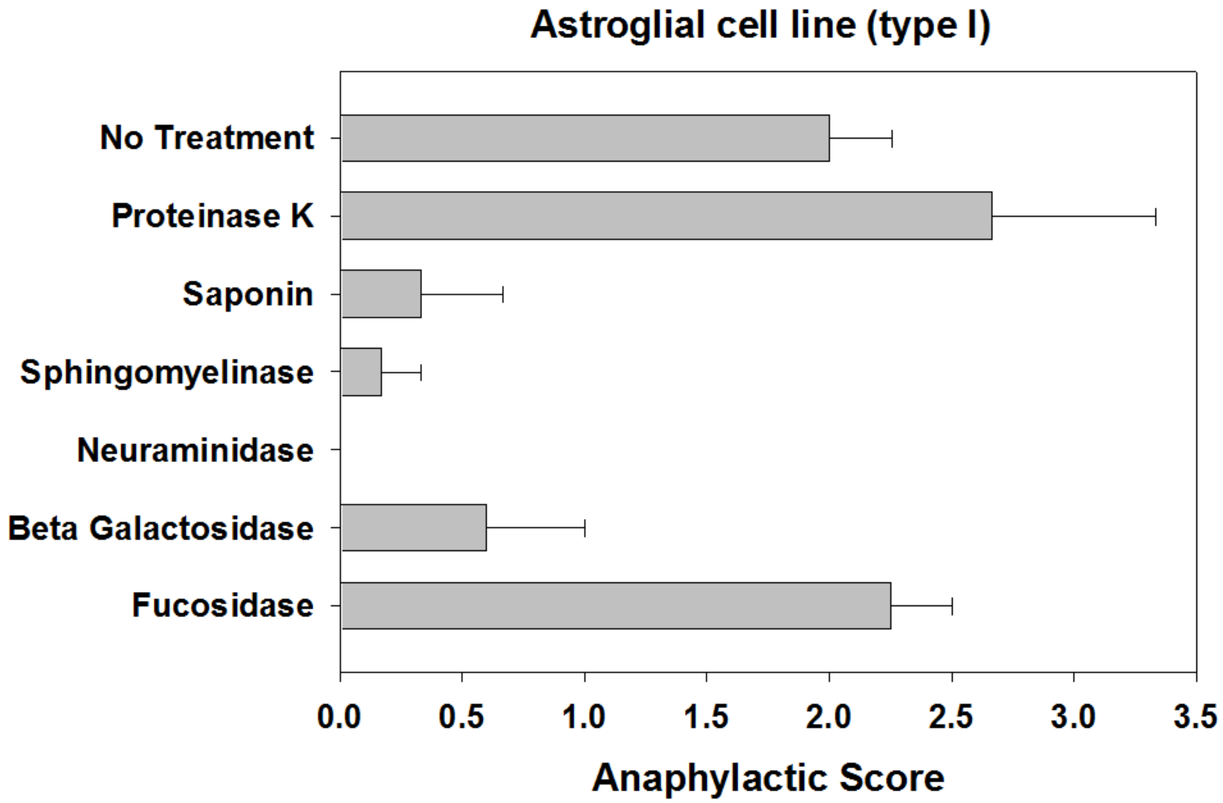

Figure S3. <u>The enzymatic analysis of the composition and the ability of the lipid rafts from the astroglial cell line to cause an anaphylactic-like reaction</u>.

Lipid rafts were isolated from the astroglial C8-D1A line and treated with proteinase K, saponin, sphyngomyelinase, neuroaminidase, β-galactosidase and fucosidase. Non-treated and treated lipid rafts were injected i.v. and the anaphylactic-like reaction was assessed during a ten-minute period. The mean maximum score ± S.E. of the total number of animals of the three separate experiments with groups of 4-5 mice in each experiment is shown.

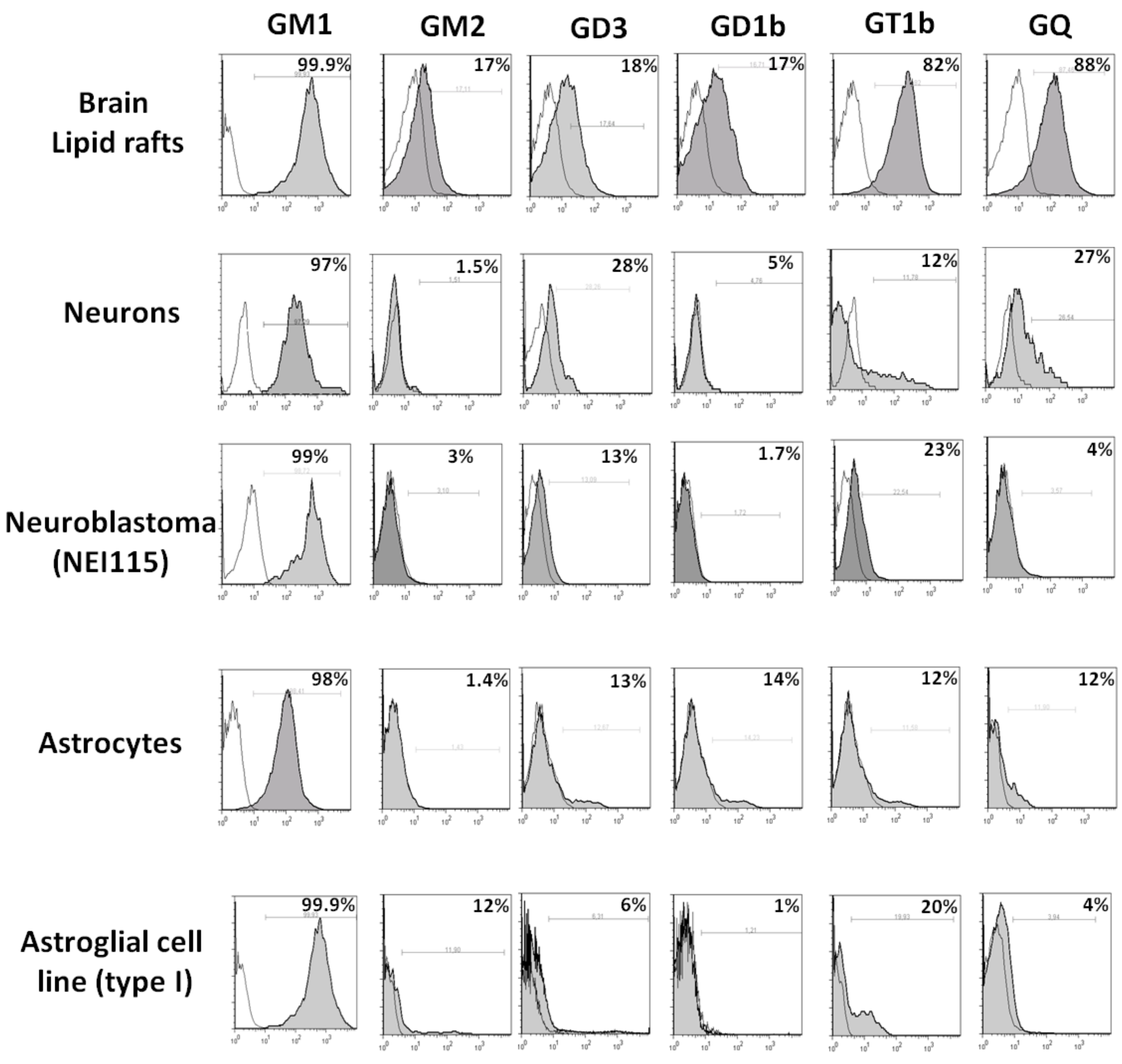

Figure S4. The <u>ganglioside expression profile of the brain lipid rafts, primary neurons, neuroblastoma cell line, primary astrocytes and astroglial cell line.</u> The suspension of brain lipid rafts, cultured neurons, neuroblastoma NIE115, cultured astrocytes, or the astroglial C8-D1A line was stained for GM1 with the fluorescently labeled CTB or with antibodies to GM2, GD3, GD1b, GT1b and GQ (A5B5) as described in *Methods* and analyzed by the flow cytometry. The filled histograms show staining for the gangliosides and the open histograms show staining for the isotype control antibodies or autofluorescence (for CTB).

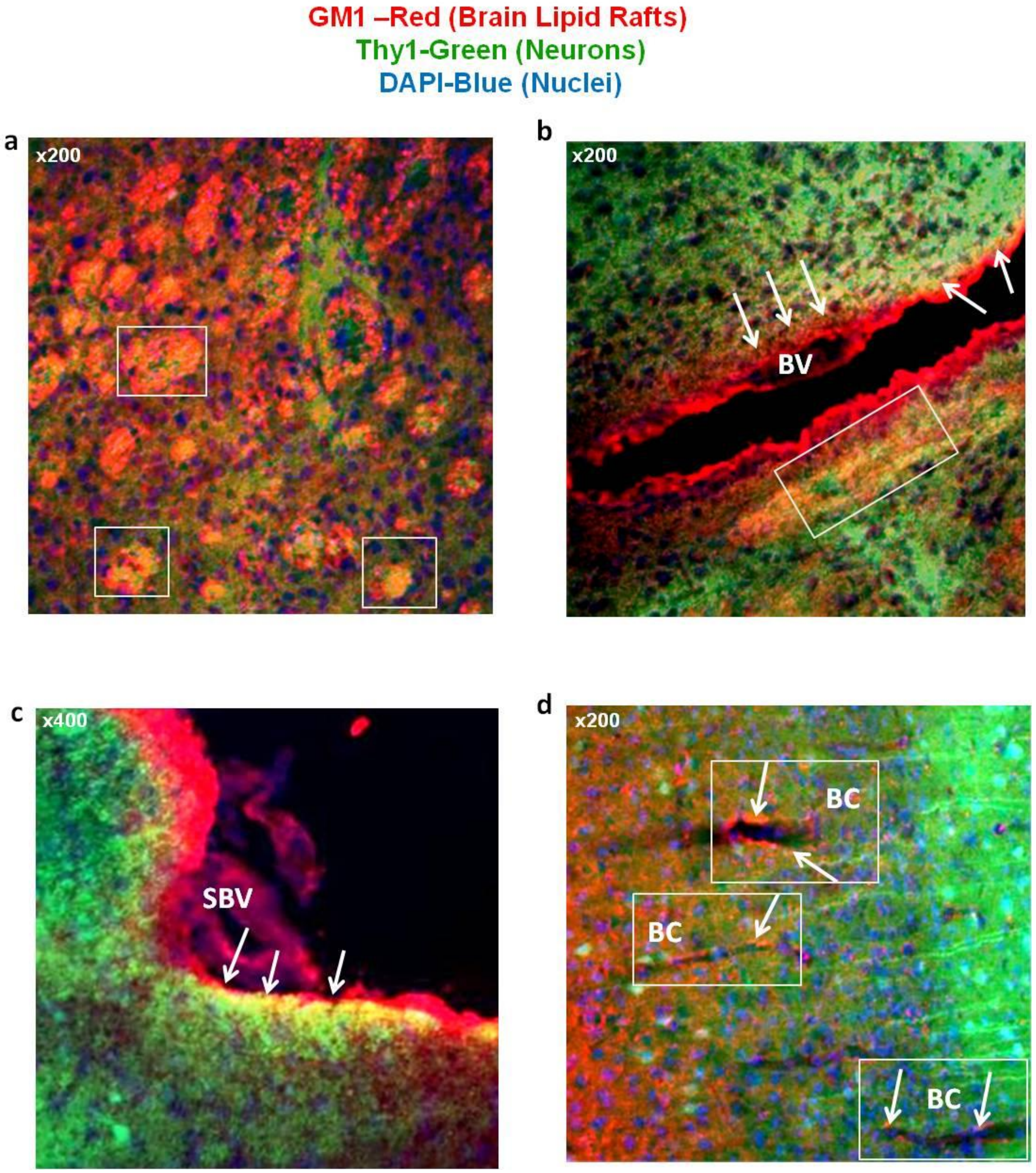

Figure S5. <u>Distribution of lipids on neuronal cells</u>. Sections of the mouse brain from Thy1-YFP reporter mice with genetically labeled neuronal bodies and axons (shown in green) were stained for GM1 with the fluorescent CTB (shown in red). Staining for the cell nuclei with DAPI is shown in blue. (**a-b**) The areas of axons that express the marker for lipid rafts GM1 are shown in yellow (co-localization of green and red colors) and marked by the rectangles. (**c-d**) The $GM1^+$ axons in close proximity to the perivascular spaces around the brain blood vessels (**b**), superficial blood vessels (**c**) or brain capillaries (**d**) are shown in yellow and marked by the arrows. The magnification for each image is shown in the upper right corner.

<u>*Abbreviations*</u>*:* BC – brain capillary, BV-blood vessel, SBV-superficial blood vessel.

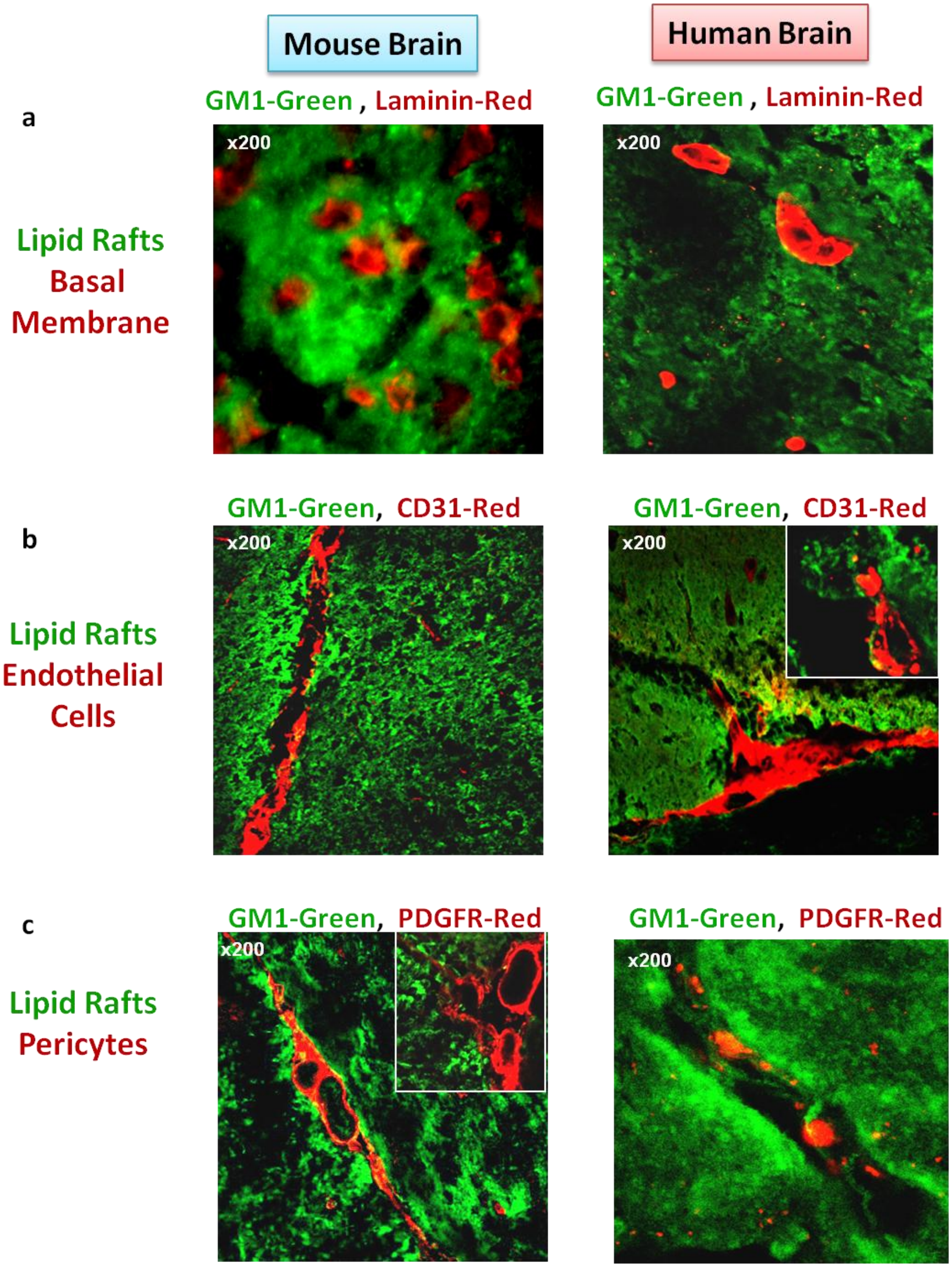

Figure S6. The <u>analysis of the lipid raft co-localization with basal membranes, endothelial cells and pericytes in the mouse and human brain</u>. Sections of the mouse (left images) or human (right images) normal brains (10µ thick) were stained for *Lipid Rafts* (**a-c**, GM1, shown in green) and for the markers for *basal membranes* (**a**, Laminin, shown in red), *endothelial cells* (**b**, CD31, shown in red) and *pericytes* (**c**, PDGFR, shown in red). In most cases, the $GM1^+$ lipid rafts were <u>not</u> co-localized with basal membranes, endothelial cells or pericytes. Additional images of the same magnification are shown in the upper right corners.

Magnification for each image is shown in the upper right corner.

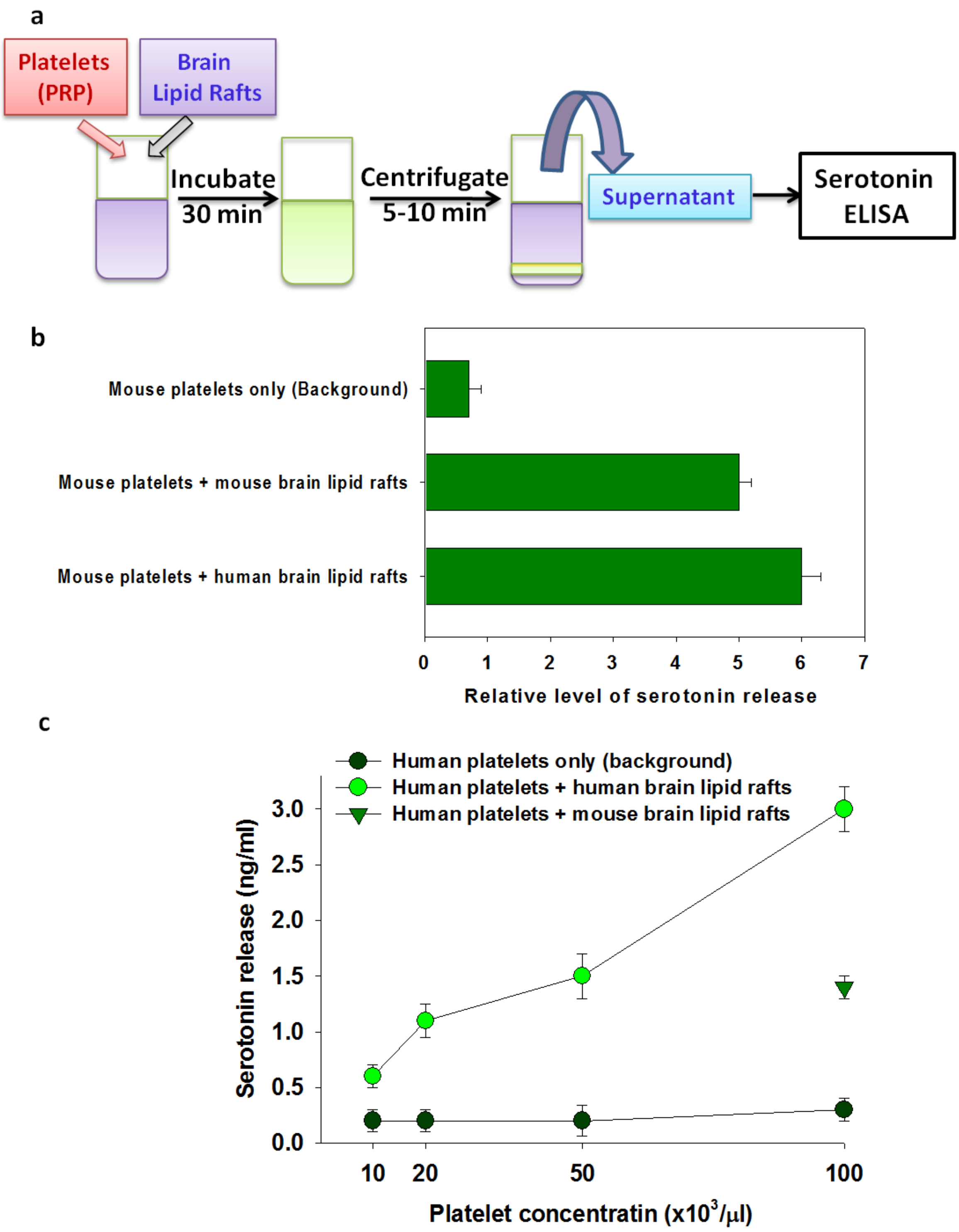

Figure S7. The <u>ability of brain lipid rafts to induce a serotonin release by mouse and human platelets *in vitro*</u>. (**a**) Brain lipid rafts were incubated with platelets, centrifuged at a high speed and the concentration of serotonin was measured in the platelet-free supernatant by ELISA as described in *Methods*. (**b,c**) The serotonin release by the mouse (**b**) or human (**c**) platelets co-incubated with the mouse or human brain lipid rafts. The mean ± S.E. of the triplicate is shown. The data are representative of the two separate experiments.

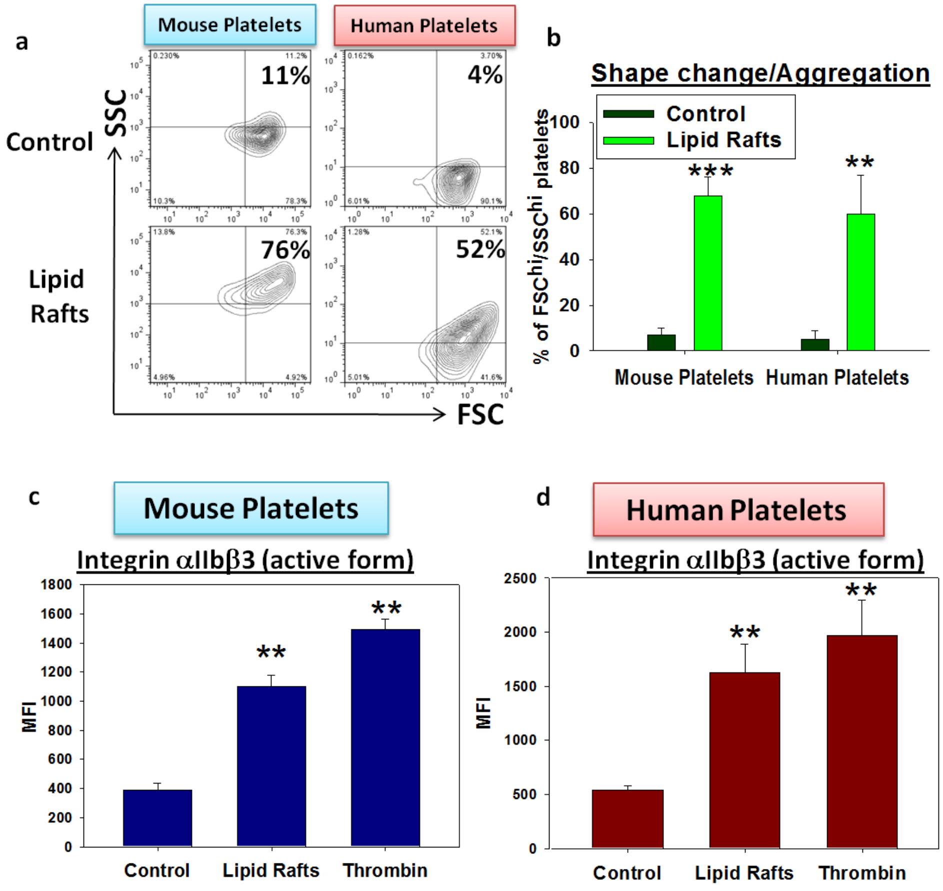

Figure S8. __**Analysis of platelet aggregation and activation marker expression after the interaction of platelets with brain lipid rafts *in vitro*.**__ (**a,b**) Mouse or human platelets were co-incubated with mouse or human brain lipid rafts as in Suppl.Fig. 7 and stained with anti-CD61 and CD41 antibodies (mouse), or anti-CD42a and CD61 antibodies (human), then analyzed by two-color flow cytometry.CD42a^{+}CD61^{+} gated human or CD41^{+}CD61^{+} gated mouse platelets were analyzed for the forward/side scatter (FCS/SSC) parameters as indicators of the platelet shape change and aggregation. (**c,d**) Mouse or human platelets were co-incubated with mouse or human brain lipid rafts or thrombin (0.5U/ml of mouse and 0.1 U/ml for human) and stained with anti-CD61, CD41 and JON/A antibodies (mouse) or anti-CD42a, CD61 and PAC-1 antibodies (human) and analyzed by three-color flow cytometry for the expression of the active form of αIIbβ3 integrin as a marker of the mouse (**c**) and human (**d**) platelet activation. In (**c,d**), thrombin was used as a positive control for the platelet activation. The mean ± S.E. of a group of 7-10 animals or healthy individuals is shown in (**b,c,d**). The percentages of aggregated (FCS^{hi}/SSC^{hi}) platelets (**b**) and the levels of expression of the active form of αIIbβ3 integrin on the platelets treated with lipid rafts or thrombin (**c,d**) were significantly higher when compared with the control group (**, $p<0.01$; ***, $p<0.001$). To exclude the possibility that the analysis of αIIbβ3 expression was influenced by a platelet aggregation, we reanalyzed our samples by gating on FCS^{low}/SSC^{low} subset and gating out the aggregated platelets based on FSC-H/FSC-W parameters. We found that the activated platelets that did not form aggregates also upregulated the active form of αIIbβ3 by 3-5 fold.

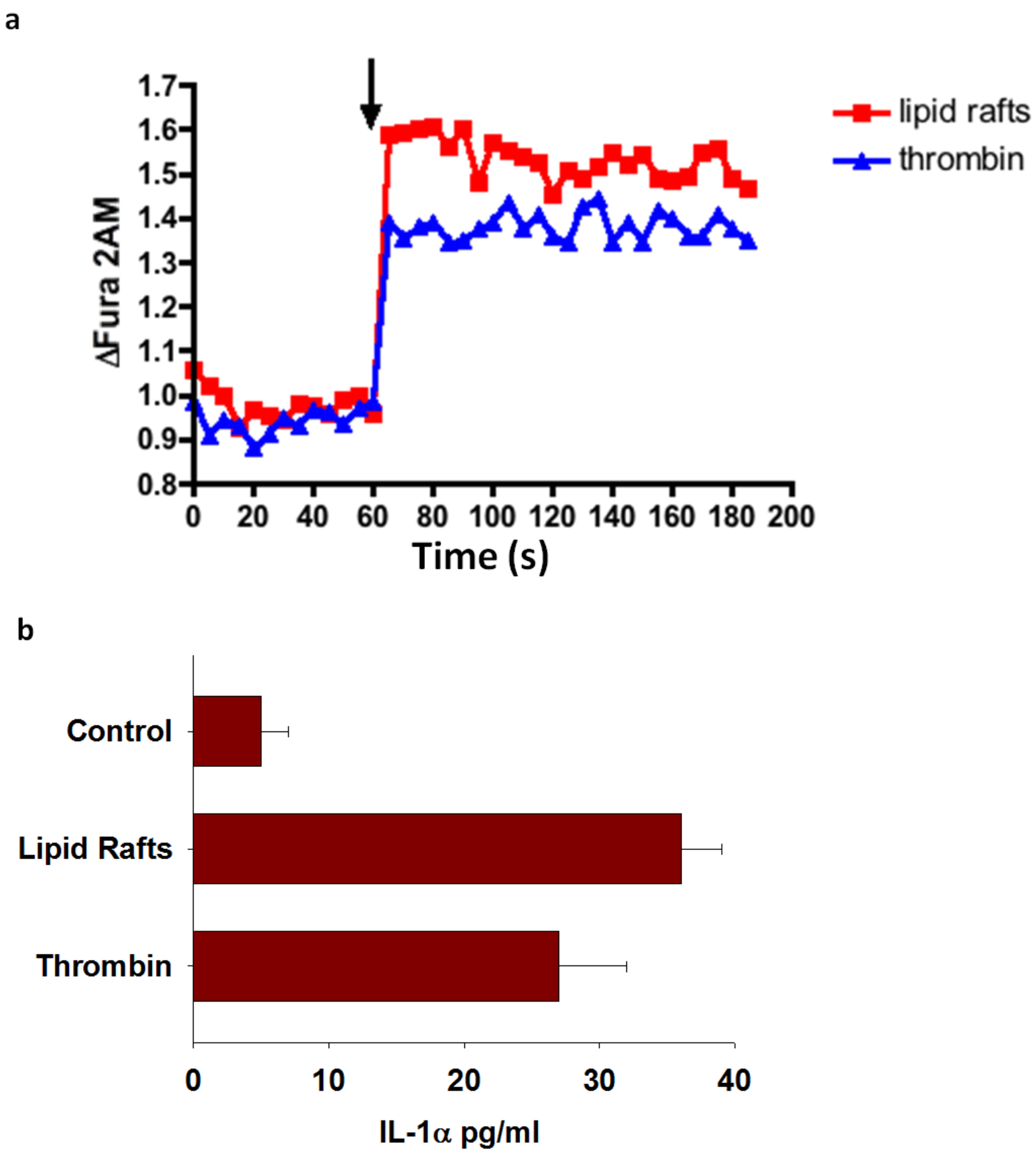

Figure S9. <u>Analysis of platelet Ca^{2+} influx and secretion of IL-1α by platelets after the interaction with brain lipid rafts</u>.

(**a**) Mouse platelets were co-incubated with brain lipid rafts or thrombin as in Suppl.Fig. 8 and the concentration of intracellular Ca^{2+} was measured using a Fura 2M fluorescent probe and TECAN spectrofluorometer. The arrow indicates the time when the lipid rafts or thrombin were added to the platelets.

(**b**) Mouse platelets were co-incubated with brain lipid rafts and the concentration of IL-1α in the platelet free supernatant was measured by ELISA as described in *Methods*.

In (**a,b**) one representative experiment of the three is shown.

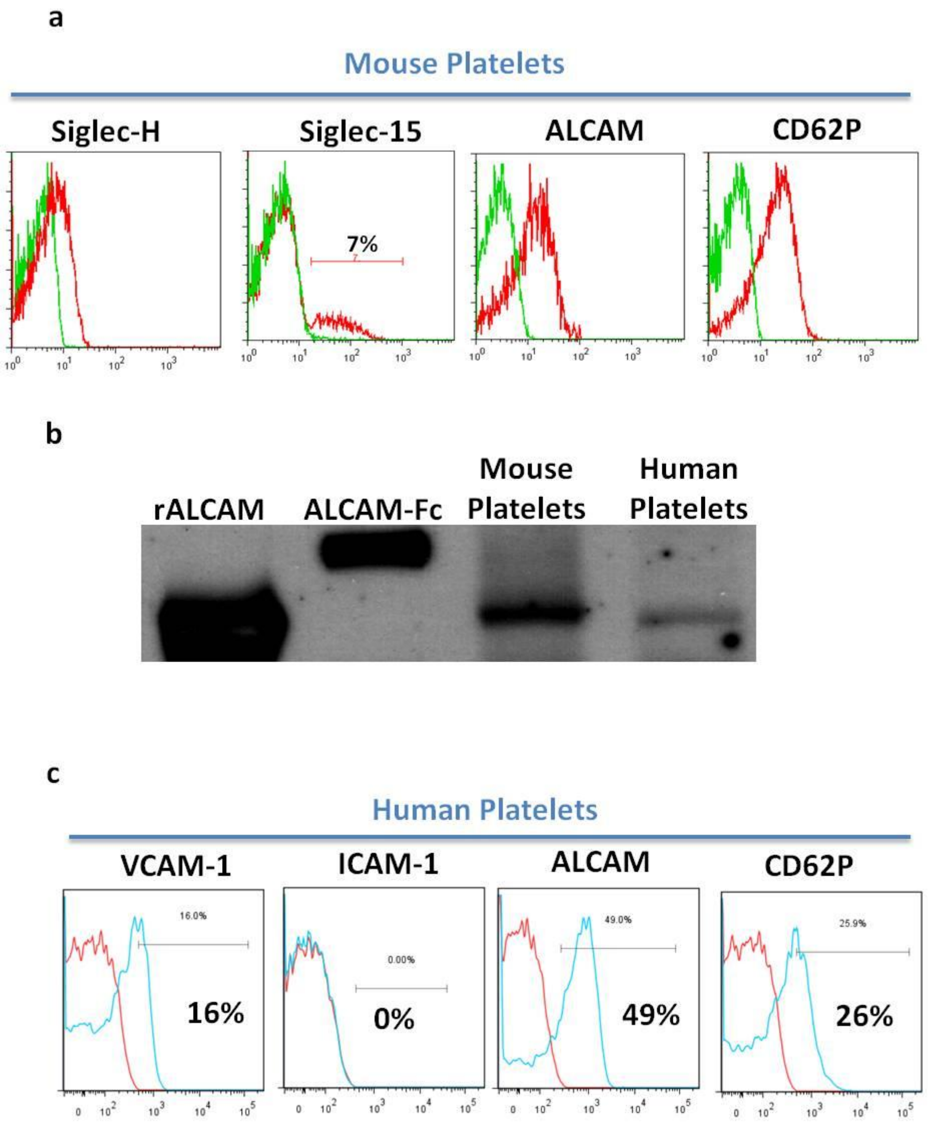

Figure S10. <u>**Analysis of the expression of siglecs and adhesion molecules in mouse and human platelets**</u>

(**a**) Flow cytometry analysis of the expression of Siglec-H, Siglec-15 and CD166 (ALCAM) and CD62P on $CD41^{+}CD61^{+}$ gated platelets. Platelets from platelet rich plasma (PRP) were stained for CD41, CD61 and Siglec-H, or Siglec-15, or CD166 and analyzed by three-color flow cytometry and described in *Methods*.

(**b**) Western-blot analysis for the expression of ALCAM in mouse and human platelets. Recombinant ALCAM protein (rALCAM, line#1) and ALCAM-Fc fusion protein (ALCAM-Fc, line#2) were used as positive controls and platelets from normal B6 mice (Mouse platelets, line#3) or normal healthy individuals (Human platelets, line#4) were used to analyze ALCAM expression in mammalian platelets.

(**c**) Platelet rich plasma was prepared from normal healthy donors and stained for CD42a and for VCAM-1, or ICAM-1, or ACAM, or CD62P and analyzed by two color flow cytometry. The percentages of positive $CD42a^{+}$ gated platelets with overlays of CD42a gated negative controls are shown.

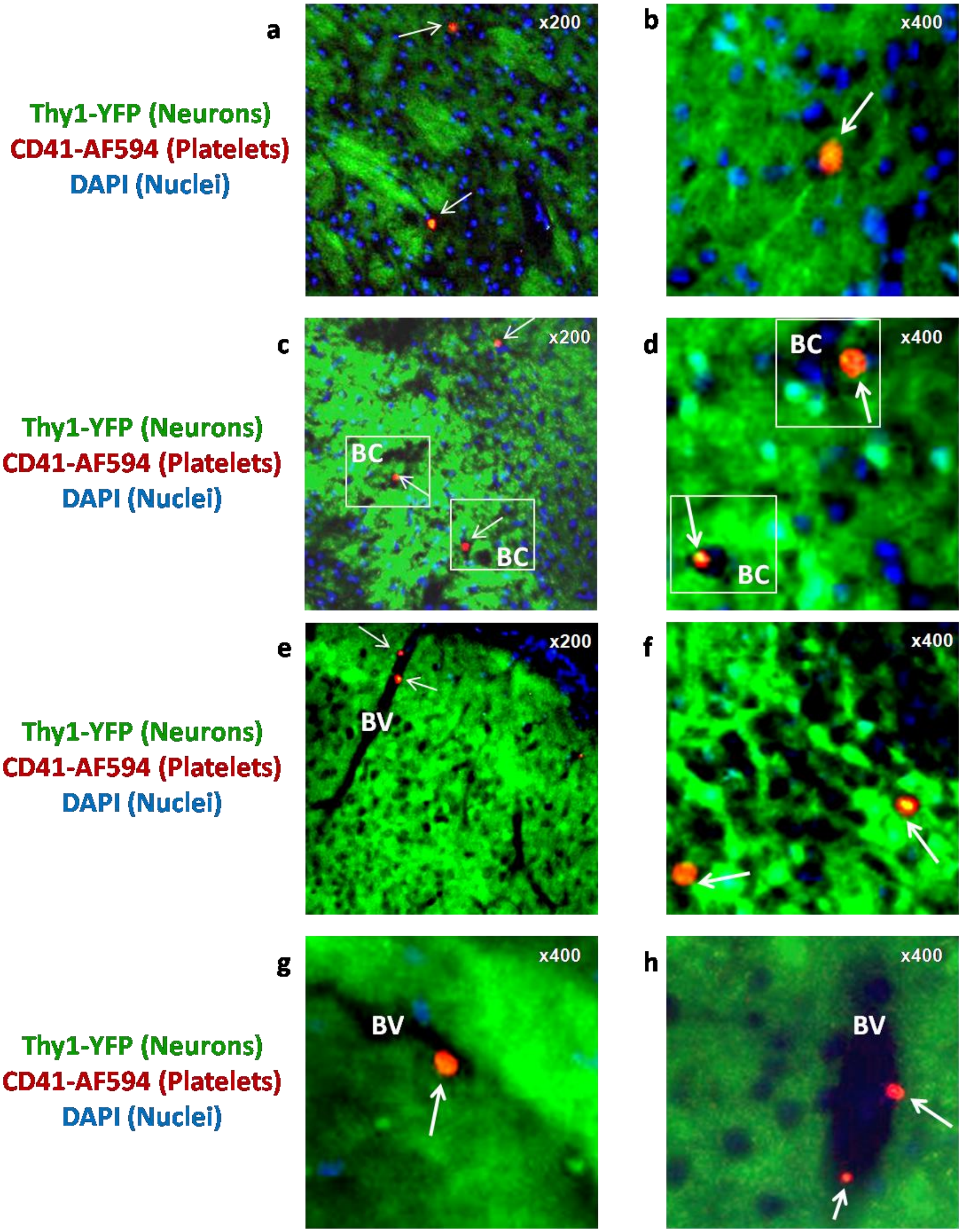

Figure S11. **<u>Co-localization of platelets with neuronal cells in the CNS of mice with EAE on day six after the disease induction</u>.** Sections of mouse brain from Thy1-YFP reporter mice with EAE (d6) with genetically labeled neuronal bodies and axons (shown in green) were stained for the platelet marker CD41 (shown in red) as described in *Methods*. Staining for the cell nuclei with DAPI is shown in blue. The Platelets (shown in red) are co-localized with neuronal axons (**a**,**b**,**h**), neuronal bodies (**c**,**f**) or both (**d**,**e**,**g**,**h**) (shown in green) and are marked by the arrows. Platelets are located in close proximity to perivascular spaces around brain blood vessels (**e**,**g**,**h**) and capillaries (**c**,**d**) and less frequently in brain parenchyma (**a**,**b**,**f**). Magnification for each image is shown in the upper right corner. *<u>Abbreviations</u>:* BC – brain capillary, BV-blood vessel.

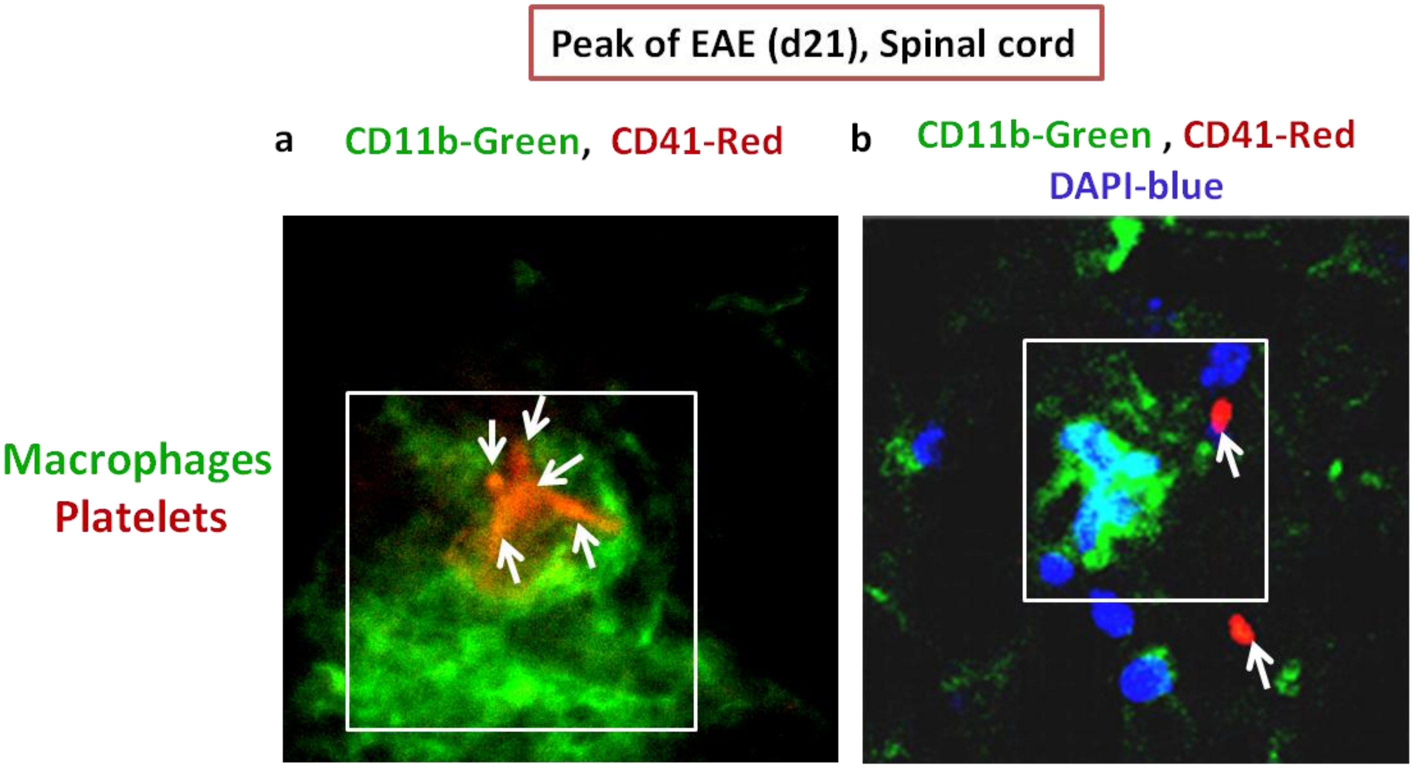

Figure S12. **<u>Localization of platelets close to inflammatory lesions in the spinal cord during the peak of EAE.</u>** EAE was induced as in Fig. 6 and on day 21 post-immunization the sections of the spinal cords of mice with EAE were stained for macrophages with CD11b (shown in green) and for platelets with CD41 (shown in red). The lesions were identified as an accumulation of $CD11b^+$ macrophages/activated microglia and indicated on the images by rectangles. $CD41^+$ platelets are indicated by arrows. Platelets (often aggregated) were detected inside the lesions (**a**) or scattered in the CNS parenchyma close to the inflammatory lesions (**b**). Magnification: x400.

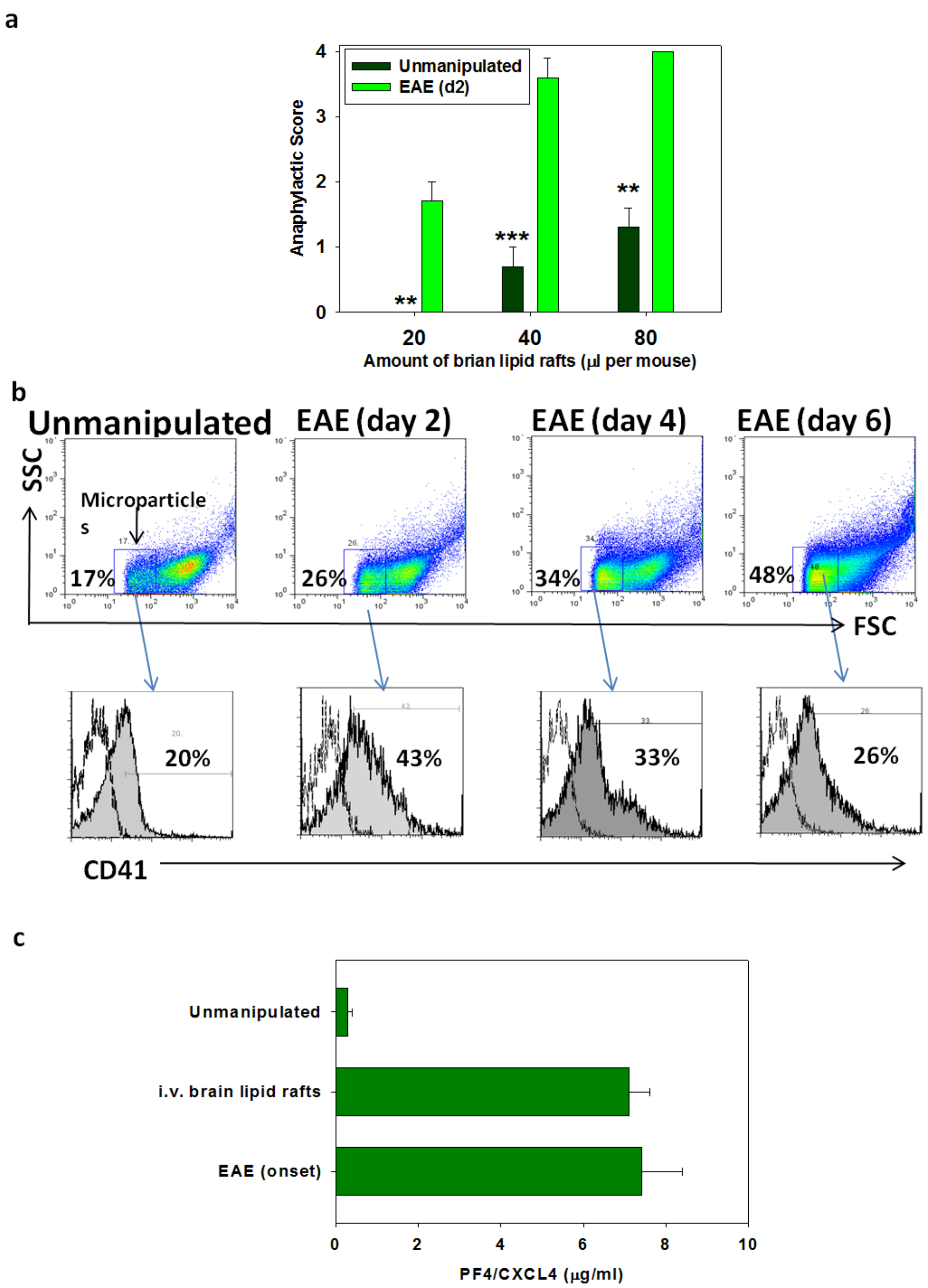

Figure S13. **Analysis of the anaphylactic reaction, platelet-derived CD41+ microparticles and PF4/CXCL4 levels in the serum of mice with EAE.** (**a**) Groups of unmanipulated mice or mice with EAE on day 2 post-immunization were injected with 20, 40 and 80 µl of brain lipid rafts per mouse as in Fig 1a. Mice were observed for a ten-minute period. Mean maximal clinical score ± S.E. of a group of 4-5 animals is shown (**, p<0.01; ***, p<0.001; unmanipulated mice vs. mice with EAE). (**b**) EAE was induced in B6 mice by immunization with MOG/CFA. PTx was injected on day 0 and day 2 post-immunization. Platelet rich plasma (PRP) was obtained from the unmanipulated mice or mice with EAE on day 2, 4 or 6 post-immunization. PRP was stained for CD41 and CD61 as described in *Methods* and analyzed by two-color flow cytometry. Forward (x-axes) and side scatter (y-axes) parameters and the percentages of events in the "microparticle" gates (sub-platelet gates) are shown in the top contour plots, and the percentages of CD41+ microparticles are shown in the bottom histograms. (**c**) Platelet free plasma was obtained from the unmanipulated mice, or from the injected mice 10 minutes post- i.v. injection of brain lipid rafts, or the mice with EAE (d6-10). The levels of the platelet factor 4 (PF4/CXCL4) expression were assessed as described in *Methods*.

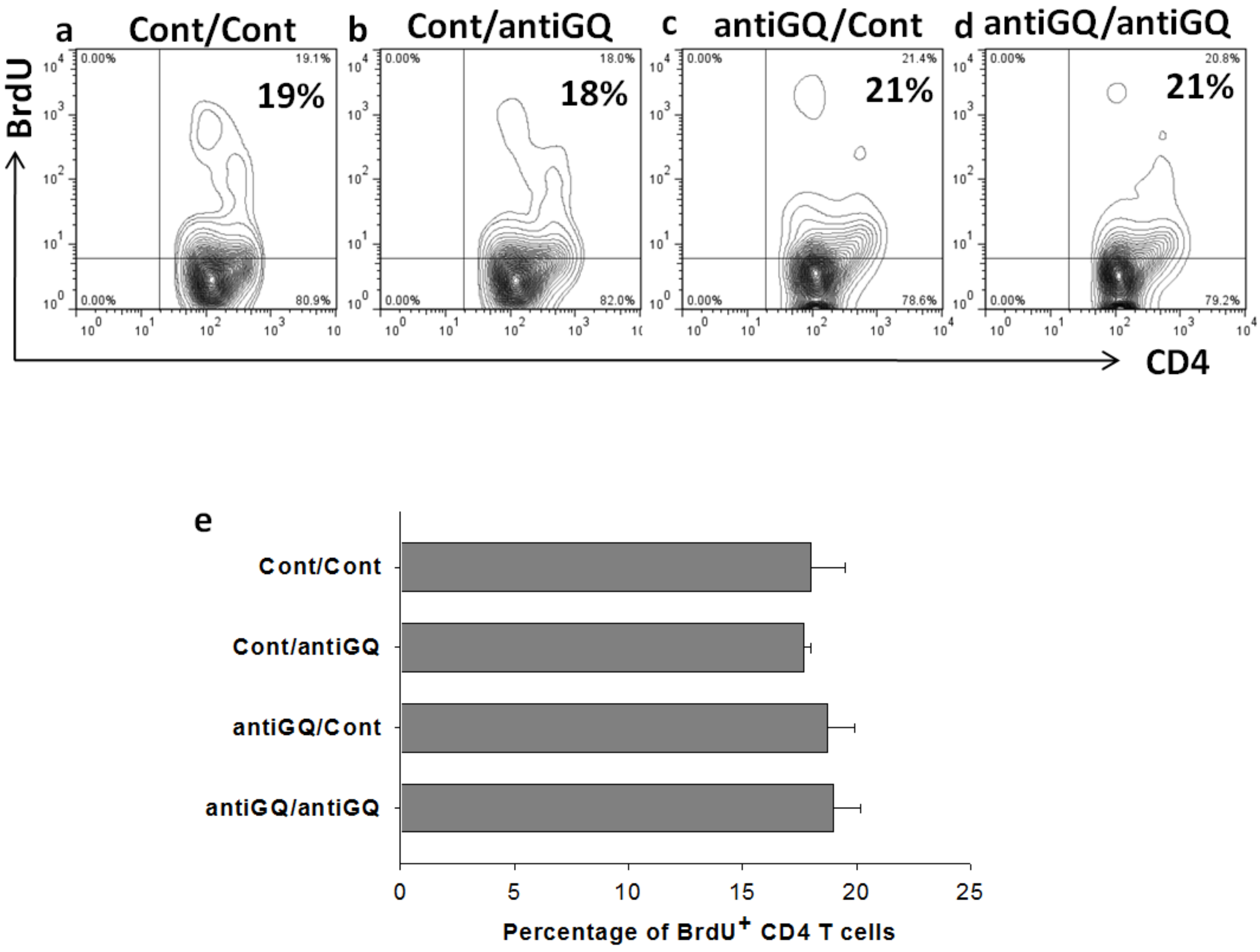

Figure S14. <u>Influence of anti-GQ (A2B5) Abs on priming and effector functions of MOG-specific autoimmune T cells.</u> MOG TCR tg 2D2 mice were immunized with MOG/CFA and PBS (**a,b**), or anti-GQ antibodies were injected on days 0, 2, 4, and 6 (**c**,**d**) as described in *Methods*. On day 7, splenocytes were isolated and CD4 T cells were purified using magnetic beads by negative selection and cultured with irradiated splenocytes for 48 hours in the presence of MOG peptide with (**b,d**) or without (**a,c**) anti-GQ Abs as described in *Methods*. To measure the cell proliferation BrdU was added 16 hrs prior to analysis. The cells were stained for CD4 (x-axis) and BrdU (y-axes), the percentage of BrdU-positive $CD4^+$ gated cells is shown in the two lower contour-plots (**a-d**). Mean ± S.E of five animals is shown in (**e**).

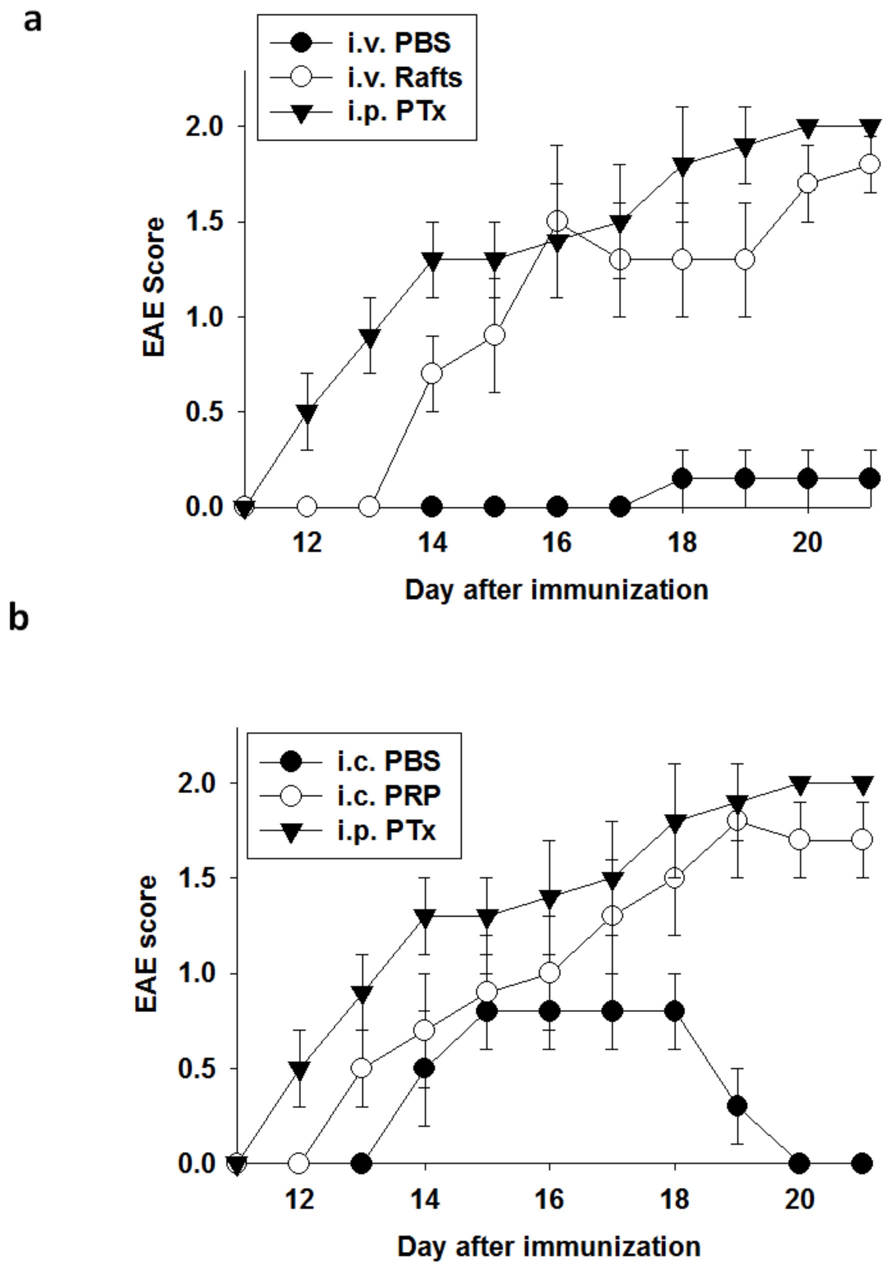

Figure S15. **<u>Induction of EAE without *Pertussis toxin* (PTx) by intravenous administration of brain lipid rafts or intracranial injection of platelets.</u>** B6 mice were immunized with MOG/CFA and on day 0 post-immunization the mice were injected i.v. with 75 μl/mouse of brain lipid rafts or PBS (**a**), or i.c. with 20 μl/mouse of platelet reach plasma (PRP), or PBS (**b**). In a separate group, PTx was administered i.p. on day 0 and day 2 post-immunization.

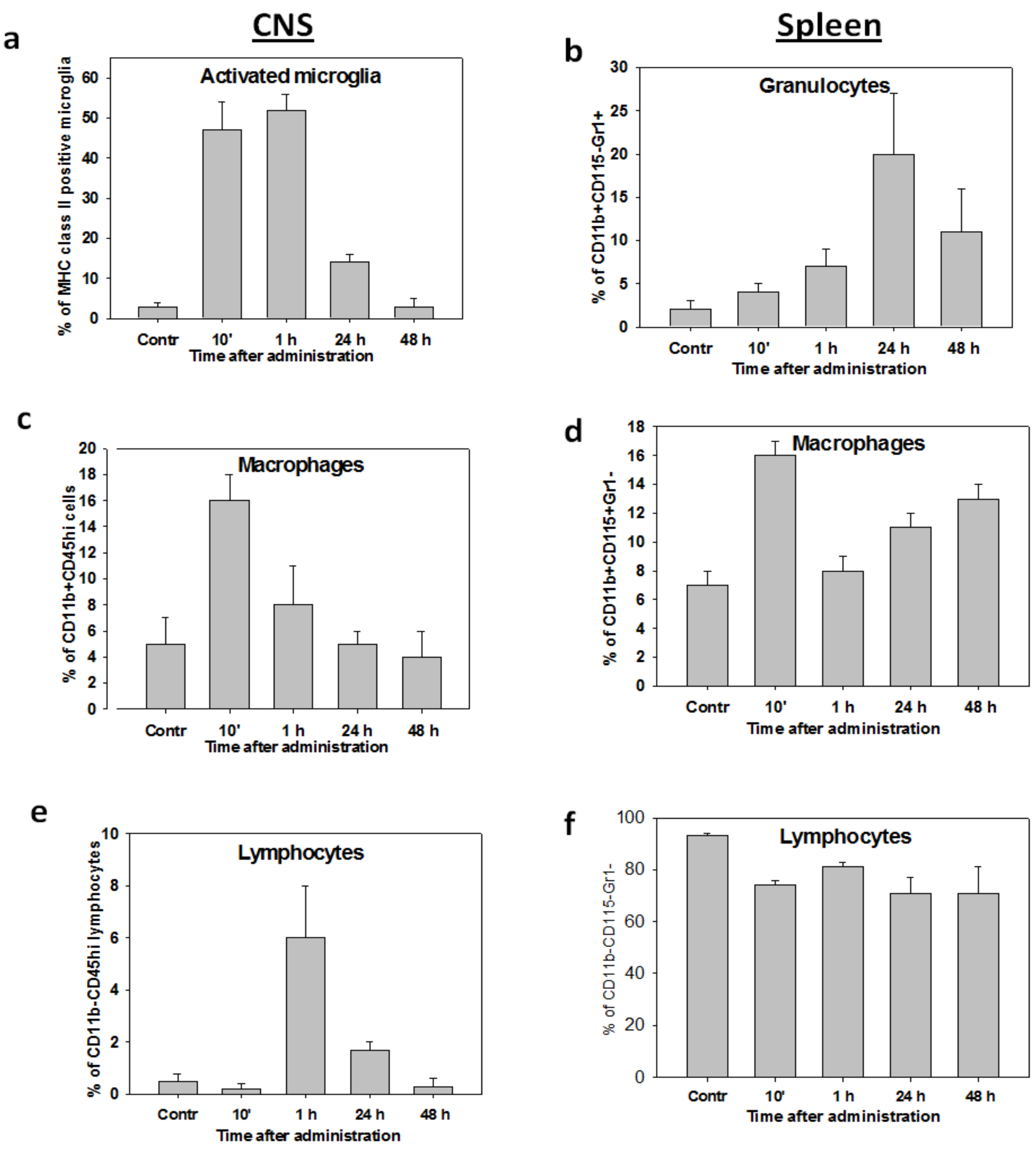

Figure S16. <u>Immune cells in the CNS and spleen following intravenous administration of brain lipid rafts.</u> Mice were injected i.v. with 75 µl of brain lipid rafts and immune cells were analyzed in the CNS and spleen at 10min, 1hr, 24hr and 48hr after the injection.

Euthanized mice were perfused with PBS and mononuclear cells from the CNS were isolated using Percoll gradient as described in *Methods*. The cells were stained for surface markers CD11b, CD45 and MHC class II and analyzed by three-color flow cytometry. Percentages of activated CD11b^{+}CD45^{low}MHC class II^{+} microglia (**a**), CD11b^{+}CD45^{hi} macrophages (**c**) and CD11b^{-}CD45^{hi} lymphocytes (**e**) are shown.

Splenocytes from the perfused mice (above) were analyzed for the expression of surface markers CD11b, CD115 (M-CSFR) and Gr1 by three-color flow cytometry. Percentages of CD11b^{+}CD115^{-}Gr1^{+} granulocytes (**b**), CD11b^{+}CD115^{+}Gr1^{-} macrophages (**d**) and CD11b^{-}CD115^{-}Gr1^{-} lymphocytes (**f**) are shown.

In **a-f**, the mean percentage ± S.E. of the total number of animals from the three separate experiments with groups of 4-5 animals is shown.

SUPPLEMENTARY DISCUSSION

Interaction of brain lipid rafts with platelets appears to be a complex process, since our data suggest that in addition to a *trans*-interaction of CD62P on platelets with brain lipid rafts (**Fig. 7A**), there may be *cis*-interactions of ALCAM and siglecs on the surface of the platelets (**Fig. 4F**). It was shown that siglecs may be blocked (or "masked") by other surface molecules such as glycosylated proteins covered by sialated carbohydrates which functionally inactivate them. This process was termed "masking" of siglecs, and the most studied example is Siglec-2 (CD22, expressed on neurons and T/B cells) and its co-receptor CD45[1]. Similar to CD45, ALCAM may mask siglecs on the platelets. ALCAM is structurally similar to a neuron- -specific NCAM (CD56) molecule and both have mono-, di- and poly- sialic acids (PSA) on the carbohydrate chains that functionally mimic several gangliosides such as GD3/GD2 and GT1b/GQ[2]. We found that ALCAM itself did not recognize sialated glycoepitopes such as 3'-sialyllactpose or α2,8-disialic acid , but could bind CD62P *in vitro* and therefore could mask P-selectin (CD62P) and other siglec receptors that are expressed on platelets (e.g. Siglec-H, Siglec-15) *in vivo*. It is supported by our findings that antibodies to ALCAM prevent the interaction of platelets with lipid rafts. In addition, ALCAM could bind a protein ligand on the brain lipid rafts as shown in **Fig. 7A**.

Our study demonstrates that an interaction of CD62P with lipid rafts depends on the presence of sialic acids on glycosilated lipids but does not require fucosylation. In addition**,** we failed to inhibit the anaphylaxis in response to the natural (as opposed to model) lipid rafts isolated from the brain by anti-ALCAM/CD62P antibodies *in vivo*. On the other hand anti-GQ antibodies inhibited the anaphylaxis caused by the natural brain lipid rafts (data not shown). These results imply that other receptors are likely to be involved in the recognition of brain lipid rafts. Potential candidates are galnectin family receptors which are known to recognize galactose (including sialated galactose)[3]. It was recently demonstrated that Galnectin-1 can recognize the GM1 ganglioside[4]; therefore, if platelets express Galnectin-1, this receptor can potentially recognize GM1 in the brain lipid rafts.

SUPPLEMENTARY REFERENCES

1. **Han, S., Collins, B.E., Bengtson, P. & Paulson, J.C. Homomultimeric complexes of CD22 in B cells revealed by protein-glycan cross-linking. *Nat Chem Biol* 1, 93-97 (2005).**
2. **Sato, C., Matsuda, T. & Kitajima, K. Neuronal differentiation-dependent expression of the disialic acid epitope on CD166 and its involvement in neurite formation in Neuro2A cells. *The Journal of biological chemistry* 277, 45299-45305 (2002).**
3. **Liu, F.T. & Rabinovich, G.A. Galectins: regulators of acute and chronic inflammation. *Annals of the New York Academy of Sciences* 1183, 158-182 (2010).**
4. **Wang, J.*, et al.* Cross-linking of GM1 ganglioside by galectin-1 mediates regulatory T cell activity involving TRPC5 channel activation: possible role in suppressing experimental autoimmune encephalomyelitis. *J Immunol* 182, 4036-4045 (2009).**
